# A Multi-Level Bayesian Analysis of Racial Bias in Police Shootings at the County-Level in the United States, 2011–2014

**DOI:** 10.1371/journal.pone.0141854

**Published:** 2015-11-05

**Authors:** Cody T. Ross

**Affiliations:** Department of Anthropology, University of California, Davis, Davis, California, United States of America; Bournemouth University, UNITED KINGDOM

## Abstract

A geographically-resolved, multi-level Bayesian model is used to analyze the data presented in the U.S. Police-Shooting Database (USPSD) in order to investigate the extent of racial bias in the shooting of American civilians by police officers in recent years. In contrast to previous work that relied on the FBI’s Supplemental Homicide Reports that were constructed from self-reported cases of police-involved homicide, this data set is less likely to be biased by police reporting practices. County-specific relative risk outcomes of being shot by police are estimated as a function of the interaction of: 1) whether suspects/civilians were armed or unarmed, and 2) the race/ethnicity of the suspects/civilians. The results provide evidence of a significant bias in the killing of unarmed black Americans relative to unarmed white Americans, in that the probability of being {black, unarmed, and shot by police} is about 3.49 times the probability of being {white, unarmed, and shot by police} on average. Furthermore, the results of multi-level modeling show that there exists significant heterogeneity across counties in the extent of racial bias in police shootings, with some counties showing relative risk ratios of 20 to 1 or more. Finally, analysis of police shooting data as a function of county-level predictors suggests that racial bias in police shootings is most likely to emerge in police departments in larger metropolitan counties with low median incomes and a sizable portion of black residents, especially when there is high financial inequality in that county. There is no relationship between county-level racial bias in police shootings and crime rates (even race-specific crime rates), meaning that the racial bias observed in police shootings in this data set is not explainable as a response to local-level crime rates.

## Introduction

In 2014, Kyle Wagner began an open contribution campaign [1] to compile all records of police-involved shootings in the United States between 2011 and 2014 in an attempt to better record the use of lethal force by police [2]. The U.S. Police-Shooting Database (USPSD) collects information on the race/ethnicity of civilians shot by police, their status as armed or unarmed, the identity of the officer(s) involved, relevant geographic information, and citations to detailed descriptions of the events.

While other databases on police shootings have been published by the government, for example through the FBI’s Supplementary Homicide Report [3], or the CDC’s National Vital Statistics System [4], these records are often censored of critical information (such as the names of the officers involved), lack independent evaluation of the justification for the shooting, and are selectively published. The FBI data, for instance, are not only incomplete, but may be structurally biased by the reporting behaviors of police, as the majority of the 17,000+ police departments in the United States do not file fatal police shooting reports, or do so only selectively [5]. According to Gabrielson et al. [5], Florida departments have failed to file reports since 1997. The data collected thus far by the USPSD help to shed light on racial bias in police shootings in Florida, which has some of the most racially-biased police shooting rates in the nation. In Miami-Dade, for example, unarmed black individuals are estimated to be more than 22 times as likely to be shot by police than unarmed white individuals. Such patterns in police violence have been immune to public scrutiny until now.

The failure of the nation’s police to critically evaluate their own use of force, has led the United Nations Committee Against Torture [6] to sharply criticize the ever growing militarization of police departments in the United States, especially as evidence of significant race-based and sexuality-based brutality and excessive use of force has been uncovered, including bonafide acts of torture (e.g., those committed by Chicago Police Commander Jon Burge and others under his command, between 1972 and 1991). The UN Committee Against Torture specifically noted that it: “regrets the lack of statistical data available on allegations of police brutality and the lack of information on the result of the investigations undertaken in respect of those allegations” (pp. 13, [6]). This paper provides a response to the first of these two concerns.

### Moving Forward

The work of documenting police violence in the United States, has recently begun through several open-contribution, public-access projects in addition to the USPSD. The Stolen Lives Project started by the Anthony Baez Foundation and the National Lawyers Guild [7], the Fatal Encounters Database started by Brian Burghart [8], and the Killed By Police database [9] are examples, as is the Mapping Police Violence project [10], which emphasizes visualization of the raw data from the above-mentioned databases. Additionally, Wikipedia.org [11], the Washington Post [12], and the Guardian [13] have begun keeping rigorous statistics on police shootings in specific years. Unlike the censored data released by official sources, the data in the USPSD and other grassroots databases allow for fine-scale evaluation of the use of lethal force, including investigation of department-specific and even officer-specific patterns. It is, for instance, possible to identify police departments and officers who kill unarmed black individuals at disproportionate rates. With the previously-used SHR data, lack of reporting and/or selective-biases in reporting of police shootings, could have masked underlying racial biases in police shootings, or masked the rates at which unarmed individuals are shot by police.

USPSD data will provide the public and federal agencies within the United States with much needed information describing where external review of police procedures, training, and practices may be needed to protect the civil rights of American citizens. Additionally, the data may be of use to: 1) communities during local elections of mayors, city council members, and police chiefs, 2) organizations, like the United Nations Committee Against Torture, reviewing allegations of racially motivated homicide and torture, and 3) academics seeking to understand the structural drivers of race-based violence and homicide by police.

#### Towards Understanding the Racial Bias in Shootings by Police

Racial bias in police shootings in the United States has been widely noted in the sociological literature for many decades [5, 14–16]. Explanations range from implicit bias in the psychology of individual officers [17–19], structural biases established by the existing social order (the conflict theory of law, and the issue of ‘minority threat’ [15, 20–22]), proximate responses by police to areas of high violence and crime (community violence theory [14, 15, 23]), racial bias in profiling and encountering suspects [24], blatant racism [25–27], social dominance orientation [28], or other factors.

With the USPSD and a geo-referenced data set on racial animus [29], an additional hypothesis is tested that police officers embedded in more normatively racist cultural contexts show increased racial bias in killing of unarmed civilians. The motivation for this hypothesis comes from sociological literature on implicit racial bias in rapid responses decisions [18, 19], in the context of frequency-dependent and conformist social learning [30] and inter-ethnic interaction [31], especially when ethnicity is spuriously associated with ‘being a threat’ [32–34] in the mind of an individual with structural power, high social dominance orientation [28], and little accountability for excessive use of force.

Much of the theoretical debate on the topic of racial bias in police shootings is sidestepped until the discussion, as much of the quantitative data underlying previous attempts at theorizing may have been fundamentally biased by the behaviors of the very actors researchers hope to understand [2, 3]. Much previous research on police shootings is based on data released by police (see, for example, [15, 35–38]). The FBI [3] clearly notes the baises in the SHR, but many authors have attempted to use the data anyway, lamenting that it is the only data available with which one can answer research questions related to police killing of civilians at a finely-resolved geographic scale [15]. The USPSD aims to reinvigorate critical research on racial bias in police shootings by providing data for analysis that is not biased by the reporting behaviors of the police.

### Research Objectives

In this paper, a multi-level, Bayesian approach is used to estimate the county-level risk ratios of being shot by police as a function of the race/ethnicity of a suspect/civilian and his/her status as armed or unarmed. As the data become more complete in the coming years, this methodology can be extended to estimate the absolute risk of being shot by police. For the purposes of this paper, however, the questions are more basic:

1. Shot by Police: Armed Versus Unarmed, by Race/Ethnicity
  - What is ratio of the probability of being {black, armed, and shot by police} to the probability of being {black, unarmed, and shot by police}?
  - What is ratio of the probability of being {hispanic, armed, and shot by police} to the probability of being {hispanic, unarmed, and shot by police}?
  - What is ratio of the probability of being {white, armed, and shot by police} to the probability of being {white, unarmed, and shot by police}?
2. Armed and Shot by Police, Across Race/Ethnicity
  - What is ratio of the probability of being {black, armed, and shot by police} to the probability of being {white, armed, and shot by police}?
  - What is ratio of the probability of being {hispanic, armed, and shot by police} to the probability of being {white, armed, and shot by police}?
3. Unarmed and Shot by Police: Across Race/Ethnicity
  - What is ratio of the probability of being {black, unarmed, and shot by police} to the probability of being {white, unarmed, and shot by police}?
  - What is ratio of the probability of being {hispanic, unarmed, and shot by police} to the probability of being {white, unarmed, and shot by police}?
4. Shot by Police: Race/Ethnicity Across Armed Status
  - What is ratio of the probability of being {black, unarmed, and shot by police} to the probability of being {white, armed, and shot by police}?
  - What is ratio of the probability of being {hispanic, unarmed, and shot by police} to the probability of being {white, armed, and shot by police}?
5. County-Level Racial Bias in Police Shootings as a Function of County-Level Properties
  Using USPSD data, is county-level racial bias in police shootings associated statistically with:
  - County-level absolute population size?
  - County-level racial/ethnic composition?
  - County-level inequality (Gini)?
  - County-level median income?
  - County-level race-specific crime rates (1. aggravated assault, and 2. weapons possession)?
  - County-level norms about racism (via a proxy variable derived from use of specific racially-based expletives in Google searches [29])?

## Results

### 1. Shot by Police: Armed Versus Unarmed, by Race/Ethnicity

The median probability across counties of being {black, armed, and shot by police} is *2.79* (PCI95: 1.72, 4.92) times the probability of being {black, unarmed, and shot by police}—the symbol, PCI95, indicates the lower and upper endpoints of central 95% of the posterior density; it is the Bayesian equivalent of a confidence interval. The median probability across counties of being {hispanic, armed, and shot by police} is *3.08* (PCI95: 2.05, 5.10) times the probability of being {hispanic, unarmed, and shot by police}. The median probability across counties of being {white, armed, and shot by police} is *3.33* (PCI95: 2.40, 4.70) times the probability of being {white, unarmed, and shot by police}.

**Fig 1:**
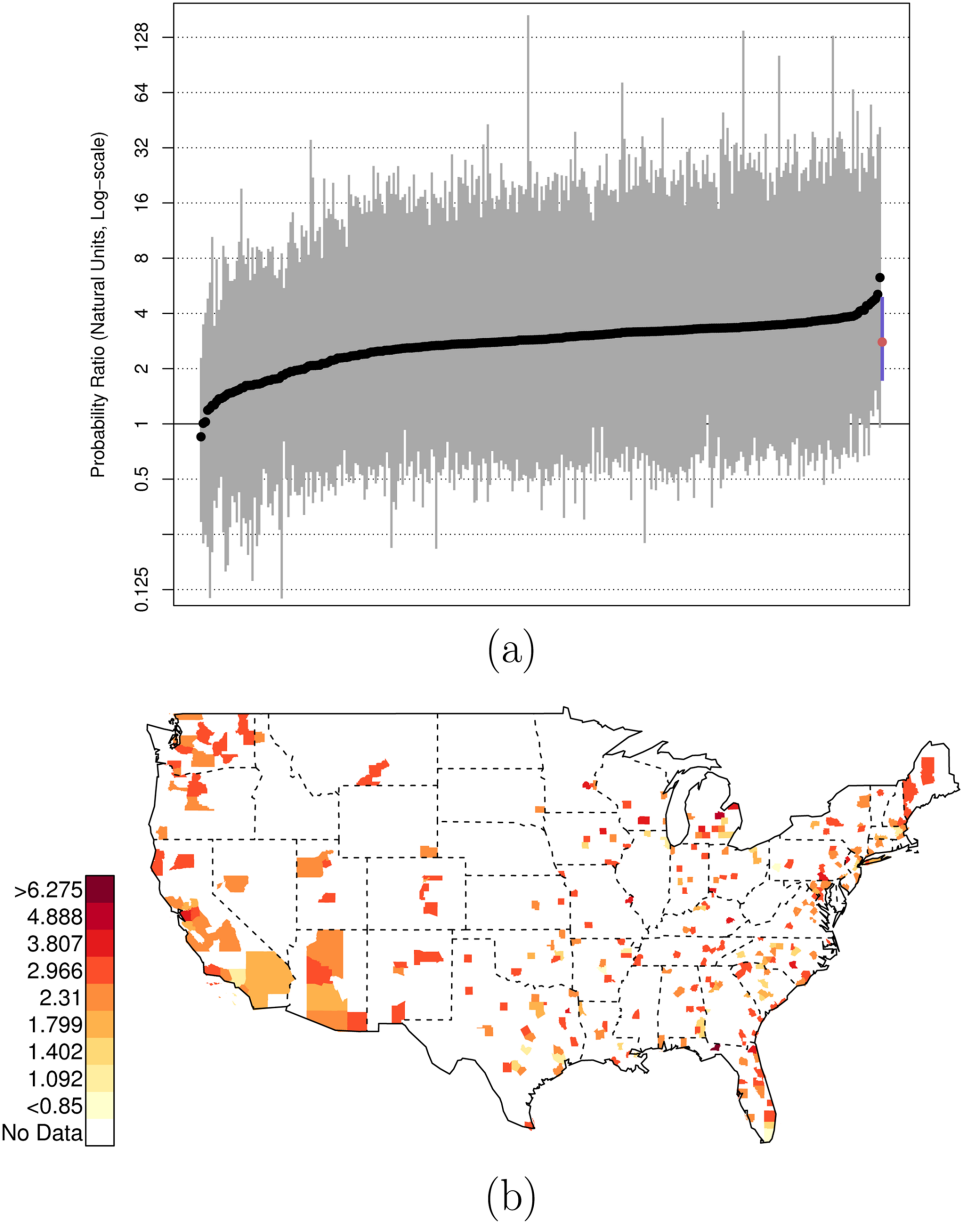
Posterior Random Effects Estimates: Risk Ratio Black, Armed-to-Unarmed. (a) County-by-county posterior estimates of the risk ratio of being {black, armed, and shot by police} to being {black, unarmed, and shot by police}. Grey bars are county-specific 95% PCI estimates. The blue bar is the nation-wide pooled 95% PCI estimate. The points on the error bars are posterior medians. Data are plotted on the log scale, but are labeled on the natural scale. (b) Map of county-specific posterior median estimates of the risk ratio of being {black, armed, and shot by police} to being {black, unarmed, and shot by police}.

**Fig 2:**
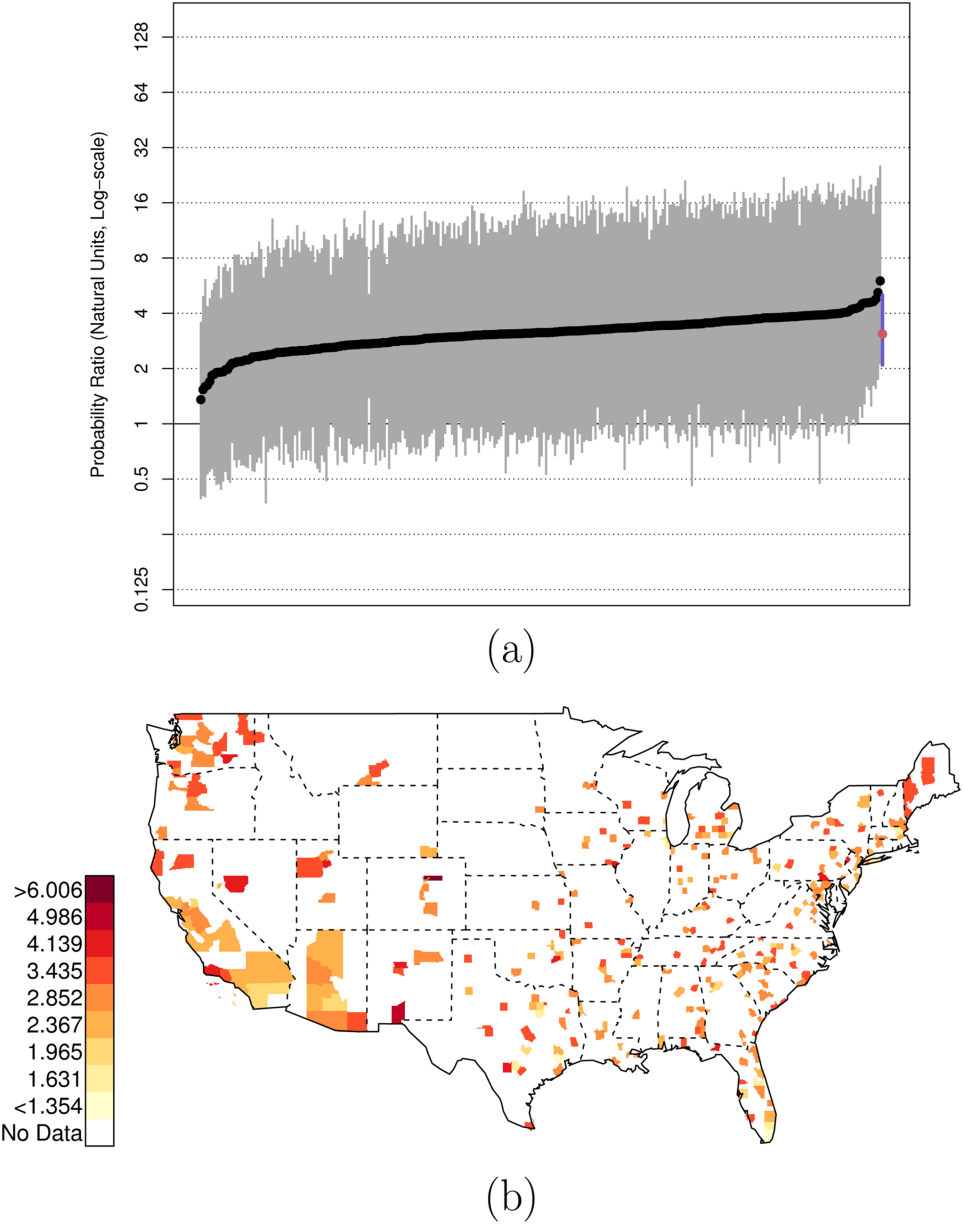
Posterior Random Effects Estimates: Risk Ratio Hispanic, Armed-to-Unarmed. (a) County-by-county posterior estimates of the risk ratio of being {hispanic, armed, and shot by police} to being {hispanic, unarmed, and shot by police}. Grey bars are county-specific 95% PCI estimates. The blue bar is the nation-wide pooled 95% PCI estimate. The points on the error bars are posterior medians. Data are plotted on the log scale, but are labeled on the natural scale. (b) Map of county-specific posterior median estimates of the risk ratio of being {hispanic, armed, and shot by police} to being {hispanic, unarmed, and shot by police}.

**Fig 3:**
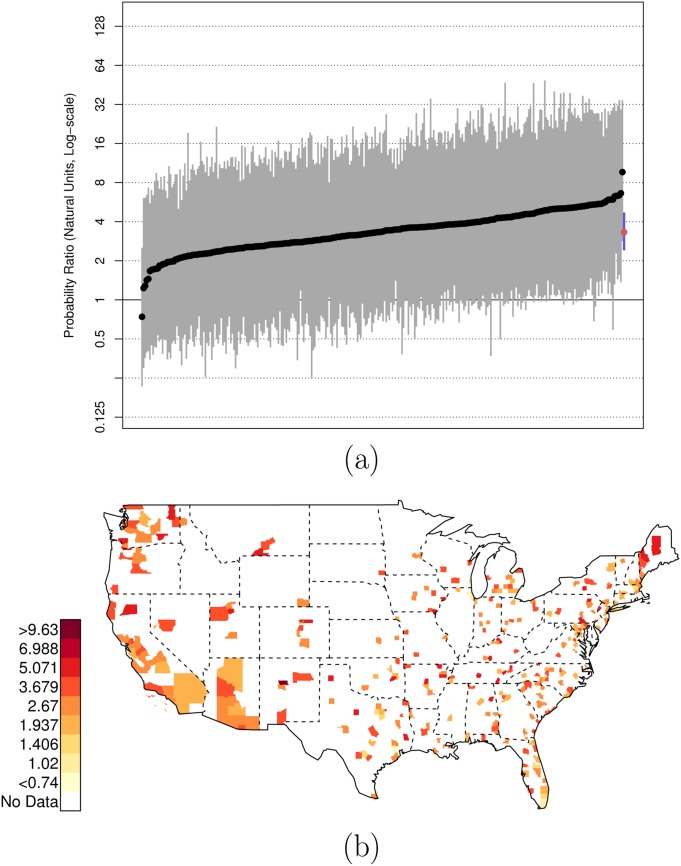
Posterior Random Effects Estimates: Risk Ratio White, Armed-to-Unarmed. (a) County-by-county posterior estimates of the risk ratio of being {white, armed, and shot by police} to being {white, unarmed, and shot by police}. Grey bars are county-specific 95% PCI estimates. The blue bar is the nation-wide pooled 95% PCI estimate. The points on the error bars are posterior medians. Data are plotted on the log scale, but are labeled on the natural scale. (b) Map of county-specific posterior median estimates of the risk ratio of being {white, armed, and shot by police} to being {white, unarmed, and shot by police}.

There is, of course, variation across counties in the U.S. in these risk ratios. Figs 1, 2, and 3 plot the posterior distributions of county-specific risk ratios, as well as the geographic distributions of the median estimates.

### 2. Armed and Shot by Police: Across Race/Ethnicity

The median probability across counties of being {black, armed, and shot by police} is *2.94* (PCI95: 2.23, 3.86) times the probability of being {white, armed, and shot by police}. The median probability across counties of being {hispanic, armed, and shot by police} is *1.57* (PCI95: 1.14, 2.09) times the probability of being {white, armed, and shot by police}.

**Fig 4:**
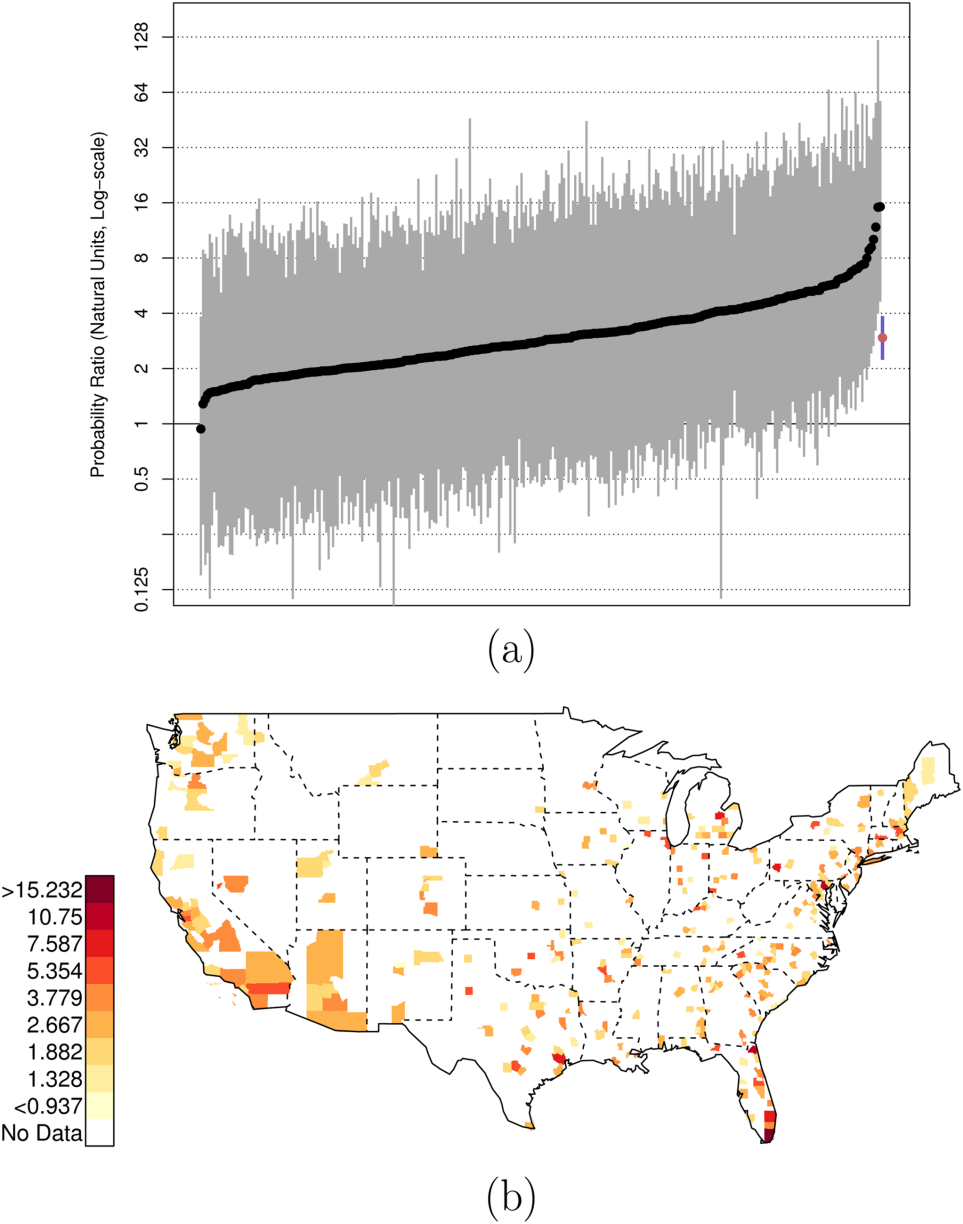
Posterior Random Effects Estimates: Risk Ratio Black-and-Armed to White-and-Armed. (a) County-by-county posterior estimates of the risk ratio of being {black, armed, and shot by police} to being {white, armed, and shot by police}. Grey bars are county-specific 95% PCI estimates. The blue bar is the nation-wide pooled 95% PCI estimate. The points on the error bars are posterior medians. Data are plotted on the log scale, but are labeled on the natural scale. (b) Map of county-specific posterior median estimates of the risk ratio of being {black, armed, and shot by police} to being {white, armed, and shot by police}.

**Fig 5:**
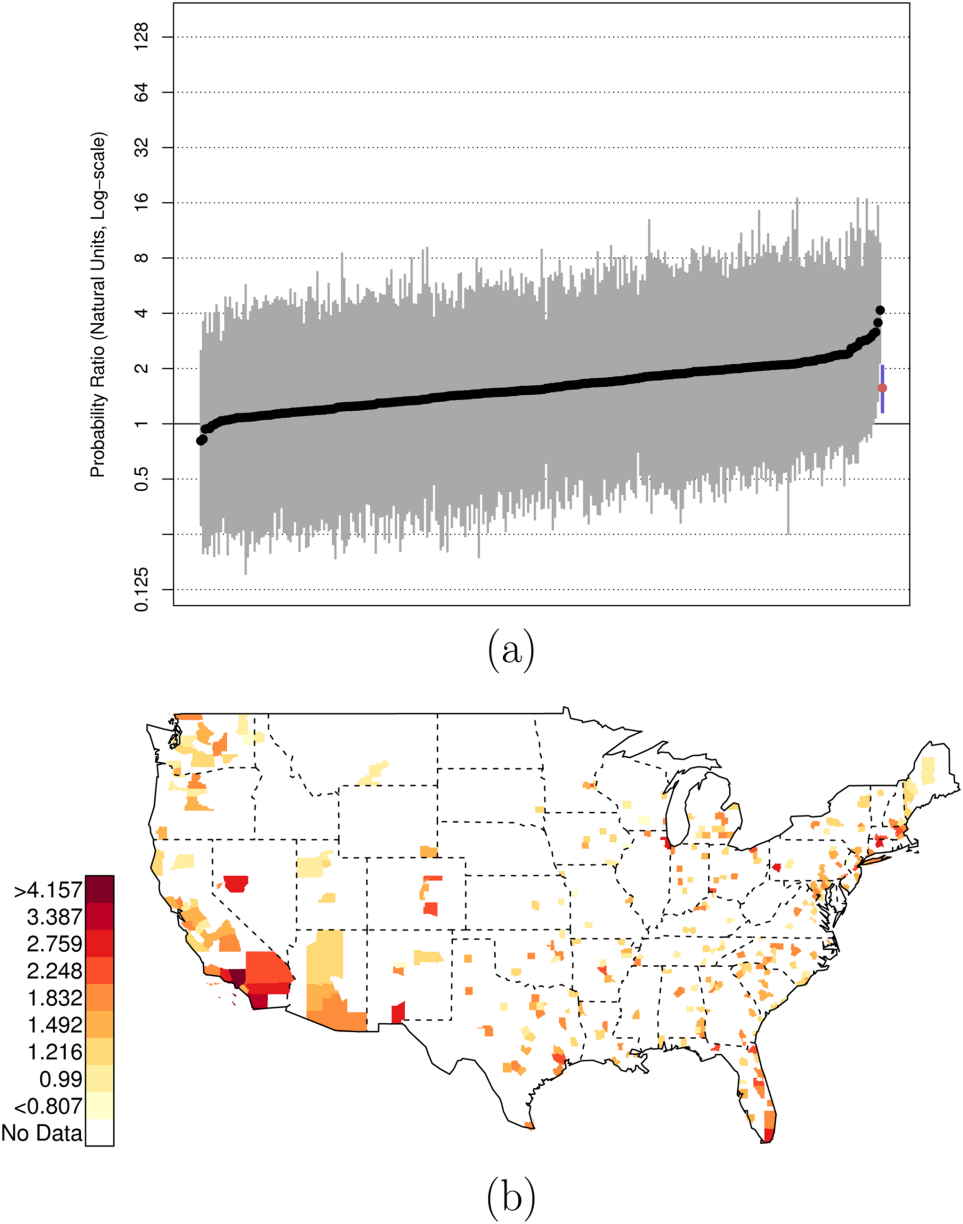
Posterior Random Effects Estimates: Risk Ratio Hispanic-and-Armed to White-and-Armed. (a) County-by-county posterior estimates of the risk ratio of being {hispanic, armed, and shot by police} to being {white, armed, and shot by police}. Grey bars are county-specific estimates. Grey bars are county-specific 95% PCI estimates. The blue bar is the nation-wide pooled 95% PCI estimate. Data are plotted on the log scale, but are labeled on the natural scale. (b) Map of county-specific posterior median estimates of the risk ratio of being {hispanic, armed, and shot by police} to being {white, armed, and shot by police}.

As before, there is variation across counties in the U.S. in these relative risk ratios. Figs 4 and 5 plot the county-specific results.

### 3. Unarmed and Shot by Police: Across Race/Ethnicity

The median probability across counties of being {black, unarmed, and shot by police} is *3.49* (PCI95: 1.77, 6.04) times the probability of being {white, unarmed, and shot by police}. The median probability across counties of being {hispanic, unarmed, and shot by police} is *1.67* (PCI95: 0.99, 2.68) times the probability of being {white, unarmed, and shot by police}.

**Fig 6:**
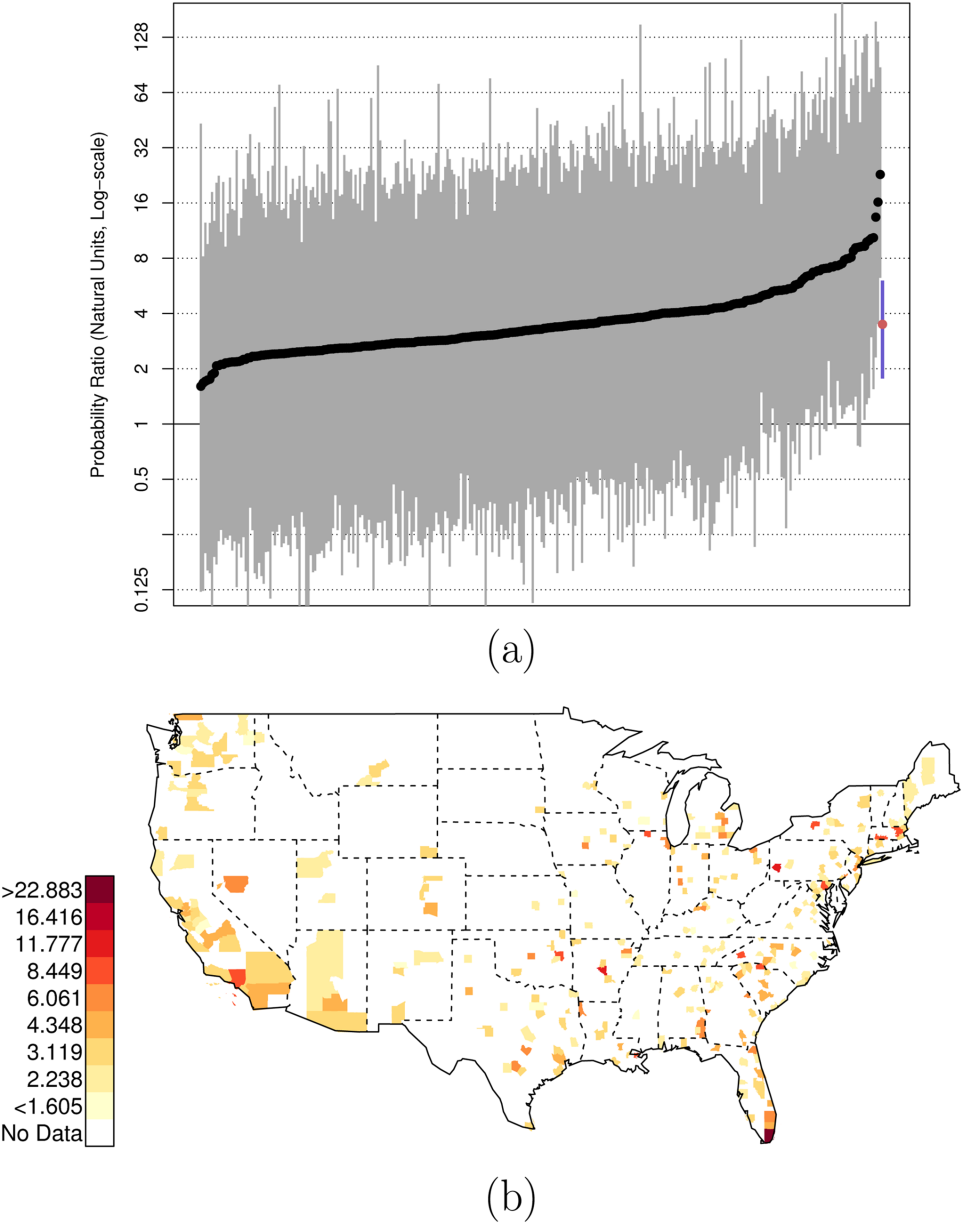
Posterior Random Effects Estimates: Risk Ratio Black-and-Unarmed to White-and-Unarmed. (a) County-by-county posterior estimates of the risk ratio of being {black, unarmed, and shot by police} to being {white, unarmed, and shot by police}. Grey bars are county-specific 95% PCI estimates. The blue bar is the nation-wide pooled 95% PCI estimate. The points on the error bars are posterior medians. Data are plotted on the log scale, but are labeled on the natural scale. (b) Map of county-specific posterior median estimates of the risk ratio of being {black, unarmed, and shot by police} to being {white, unarmed, and shot by police}.

**Fig 7:**
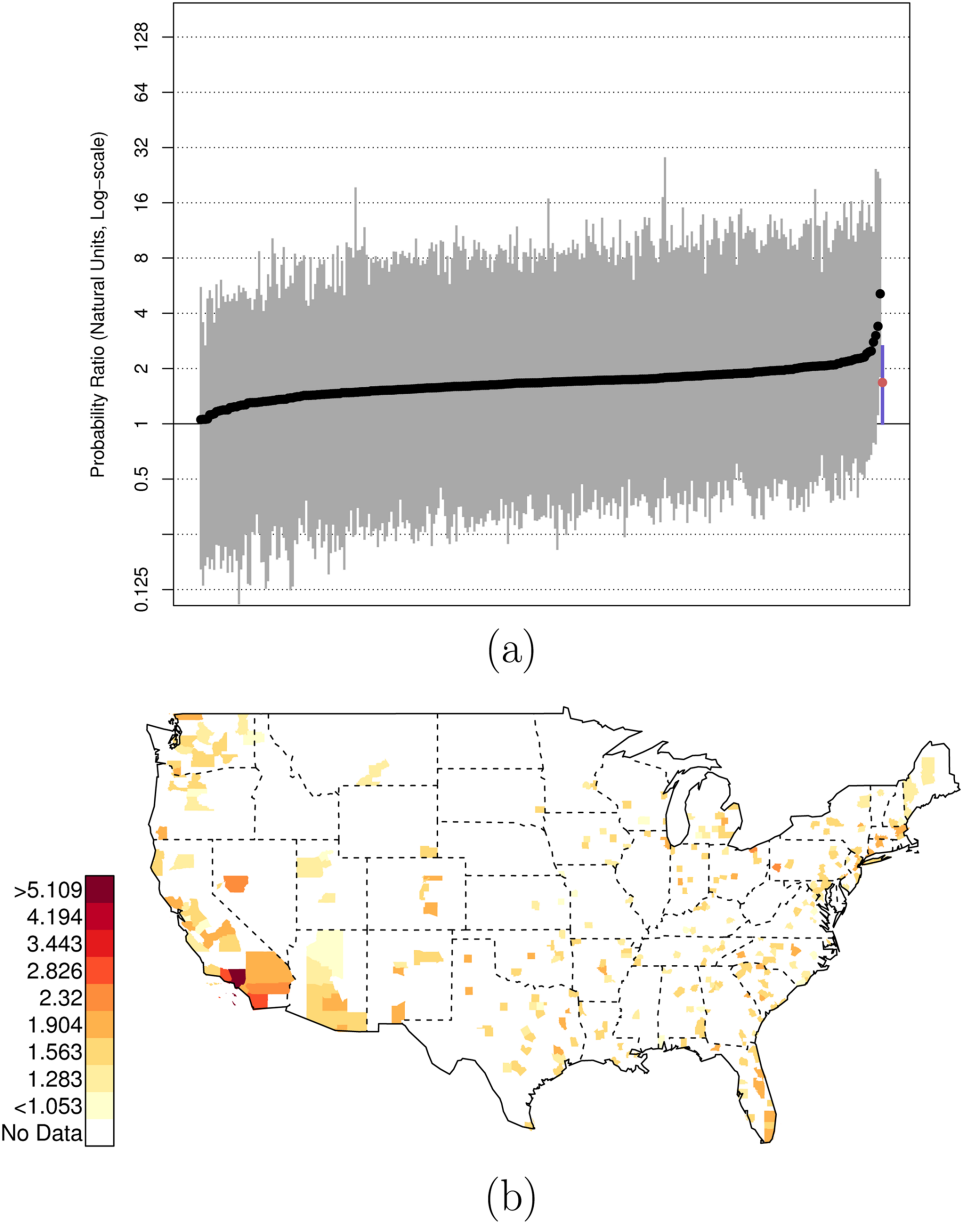
Posterior Random Effects Estimates: Risk Ratio Hispanic-and-Unarmed to White-and-Unarmed. (a) County-by-county posterior estimates of the risk ratio of being {hispanic, unarmed, and shot by police} to being {white, unarmed, and shot by police}. Grey bars are county-specific 95% PCI estimates. The blue bar is the nation-wide pooled 95% PCI estimate. The points on the error bars are posterior medians. Data are plotted on the log scale, but are labeled on the natural scale. (b) Map of county-specific posterior median estimates of the risk ratio of being {hispanic, unarmed, and shot by police} to being {white, unarmed, and shot by police}.

As before, there is extensive variation across counties in the U.S. in these relative risk ratios. Figs 6 and 7 plot the posterior distributions of county-specific risk ratios, as well as the geographic distributions of the estimates. It is notable that Miami-Dade (FL, contains Miami), Los Angeles (CA, contains Los Angeles), and Orleans Parish (LA, contains New Orleans), stand out as counties where the ratio of {black, unarmed, and shot by police} to {white, unarmed, and shot by police} is elevated to 22.88 (PCI95: 6.25, 87.70), 10.25 (PCI95: 2.96, 76.05), and 9.29 (PCI95: 1.88, 105.54) respectively. See Data folder of S1 File for additional county-level results; there are several additional counties with highly elevated levels of racial bias in police shootings not listed here.

### 4. Shot by Police: Race/Ethnicity Across Armed Status

It is worth noting, that on average across counties in the United States, an individual is as likely to be {black, *unarmed*, and shot by police} as {white, *armed*, and shot by police}, with a median relative risk estimate of 1.04 (PCI95: 0.62, 1.61). The corresponding ratio for hispanics is 0.52 (PCI95: 0.32, 0.75).

**Fig 8:**
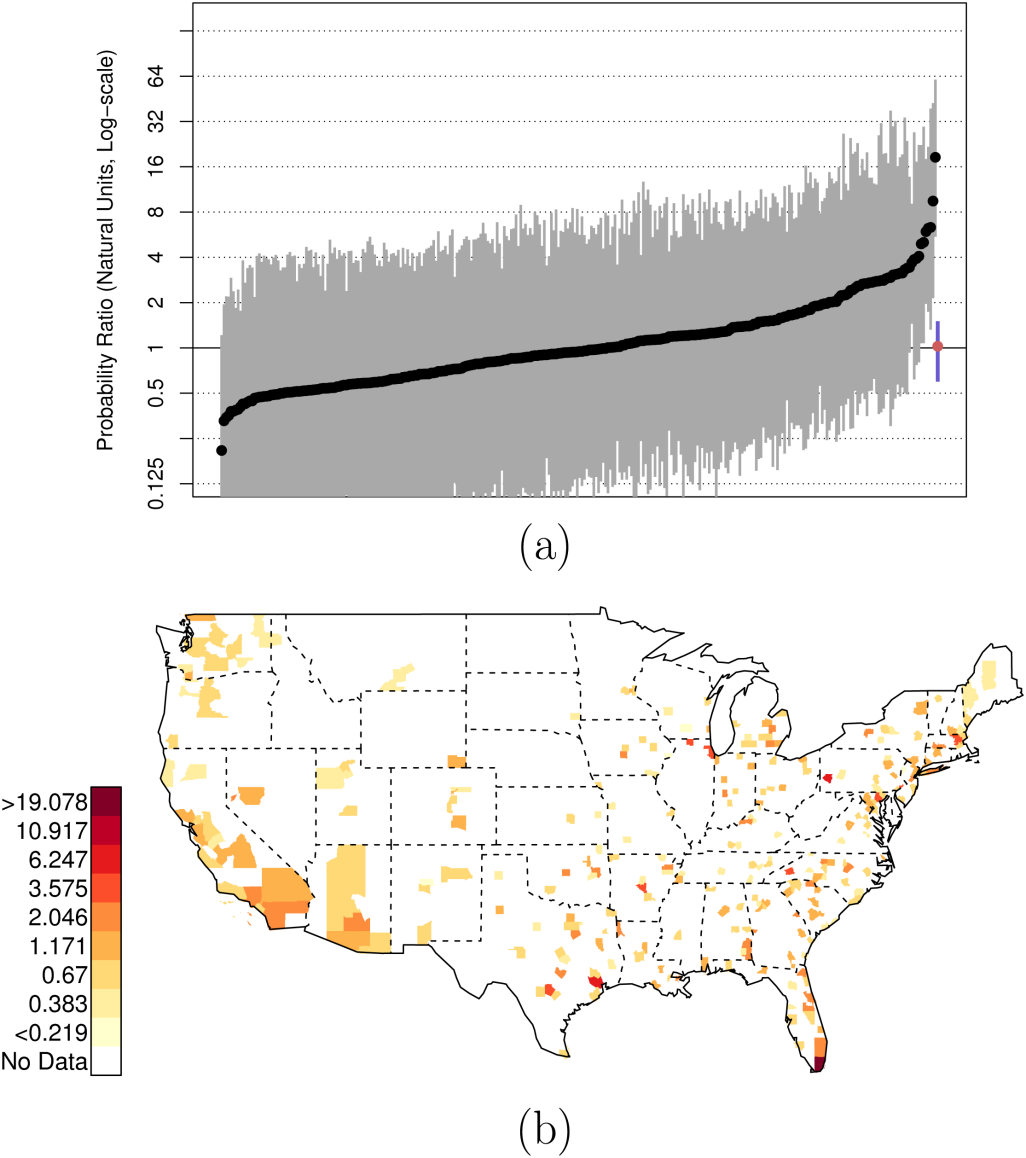
Posterior Random Effects Estimates: Risk Ratio Black-and-Unarmed to White-and-Armed. (a) County-by-county posterior estimates of the risk ratio of being {black, unarmed, and shot by police} to being {white, armed, and shot by police}. Grey bars are county-specific 95% PCI estimates. The blue bar is the nation-wide pooled 95% PCI estimate. The points on the error bars are posterior medians. Data are plotted on the log scale, but are labeled on the natural scale. (b) Map of county-specific posterior median estimates of the risk ratio of being {black, unarmed, and shot by police} to being {white, armed, and shot by police}.

**Fig 9:**
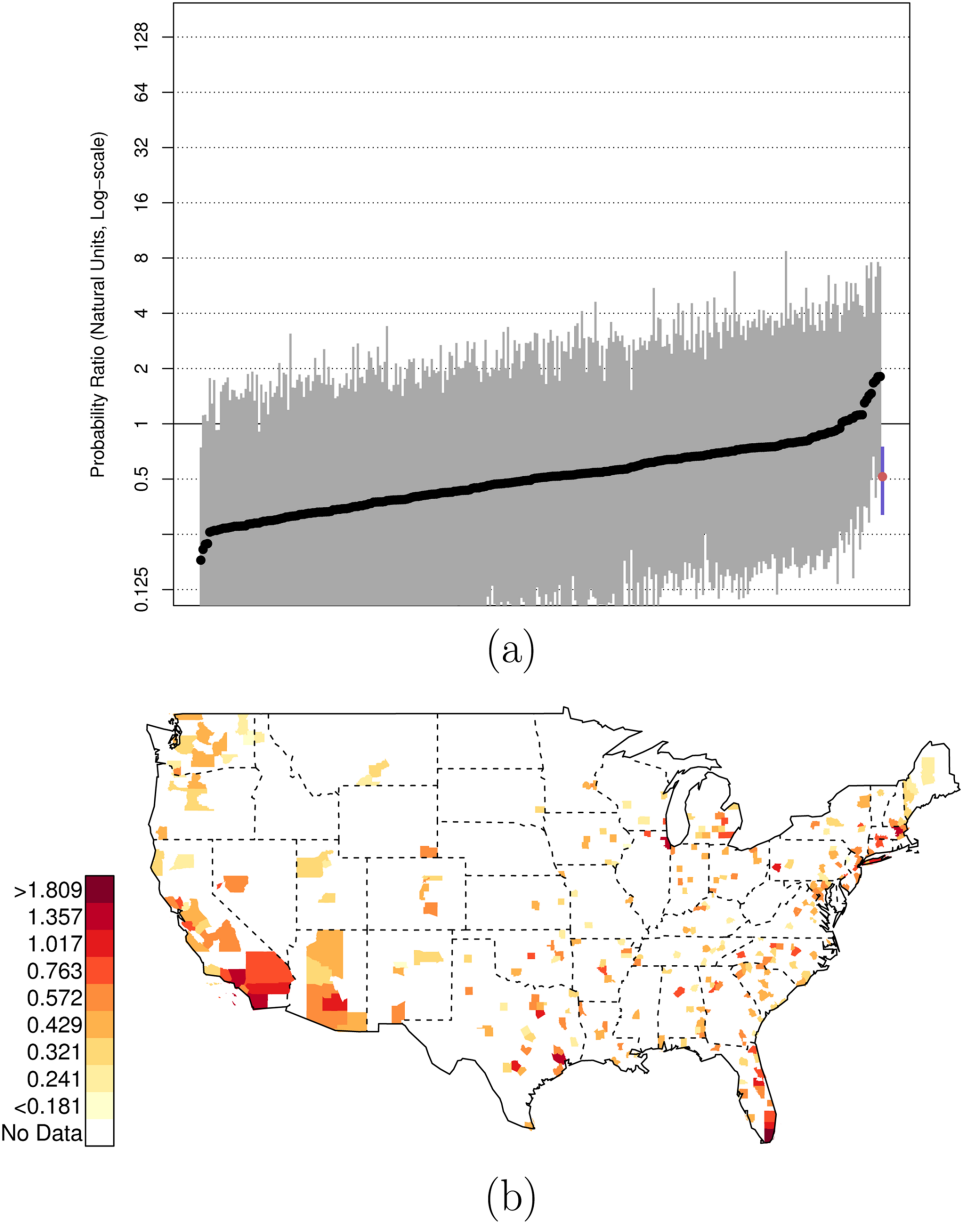
Posterior Random Effects Estimates: Risk Ratio Hispanic-and-Unarmed to White-and-Armed. (a) County-by-county posterior estimates of the risk ratio of being {hispanic, unarmed, and shot by police} to being {white, armed, and shot by police}. Grey bars are county-specific 95% PCI estimates. The blue bar is the nation-wide pooled 95% PCI estimate. The points on the error bars are posterior medians. Data are plotted on the log scale, but are labeled on the natural scale. (b) Map of county-specific posterior median estimates of the risk ratio of being {hispanic, armed, and shot by police} to being {white, unarmed, and shot by police}.

Figs 8 and 9 plot the posterior distributions of county-specific risk ratios, as well as the geographic distributions of the estimates. It is notable that Miami-Dade (FL, contains Miami), Harris (TX, contains Houston), and Cook (IL, contains Chicago), stand out as counties where the ratio of {black, unarmed, and shot by police} to {white, armed, and shot by police} is elevated to 19.08 (PCI95: 4.46, 81.13), 6.71 (PCI95: 1.46, 26.77), and 5.60 (PCI95: 1.25, 21.97) respectively. As before, the Data folder of S1 File shows that there are several other counties with highly elevated relative risk ratios in addition to those discussed above.

### 5. County-Level Racial Bias in Police Shootings as a Function of County-Level Properties

Understanding the source of racial bias in police shootings is difficult to do from county-level data, as the ecological inference fallacy can potentially obscure any results [39]. County-level data are far too coarse to use to reliably tease apart the conditions that drive racial bias in police shootings; more reliable findings will likely be based on rigorous, yet qualitative, investigations that are resolved to a more local level. Nevertheless, previous quantitative studies have used regression models on county-level data to compare theories about the county-level correlates of racial bias in police homicide [15, 35–38]. For comparability, this study uses similar models to analyze the USPSD data, contrasting the predictions from conflict and community violence theories, and testing a hypothesis about the possible association of community-level norms about racism and racial bias in police shooting.

**Fig 10:**
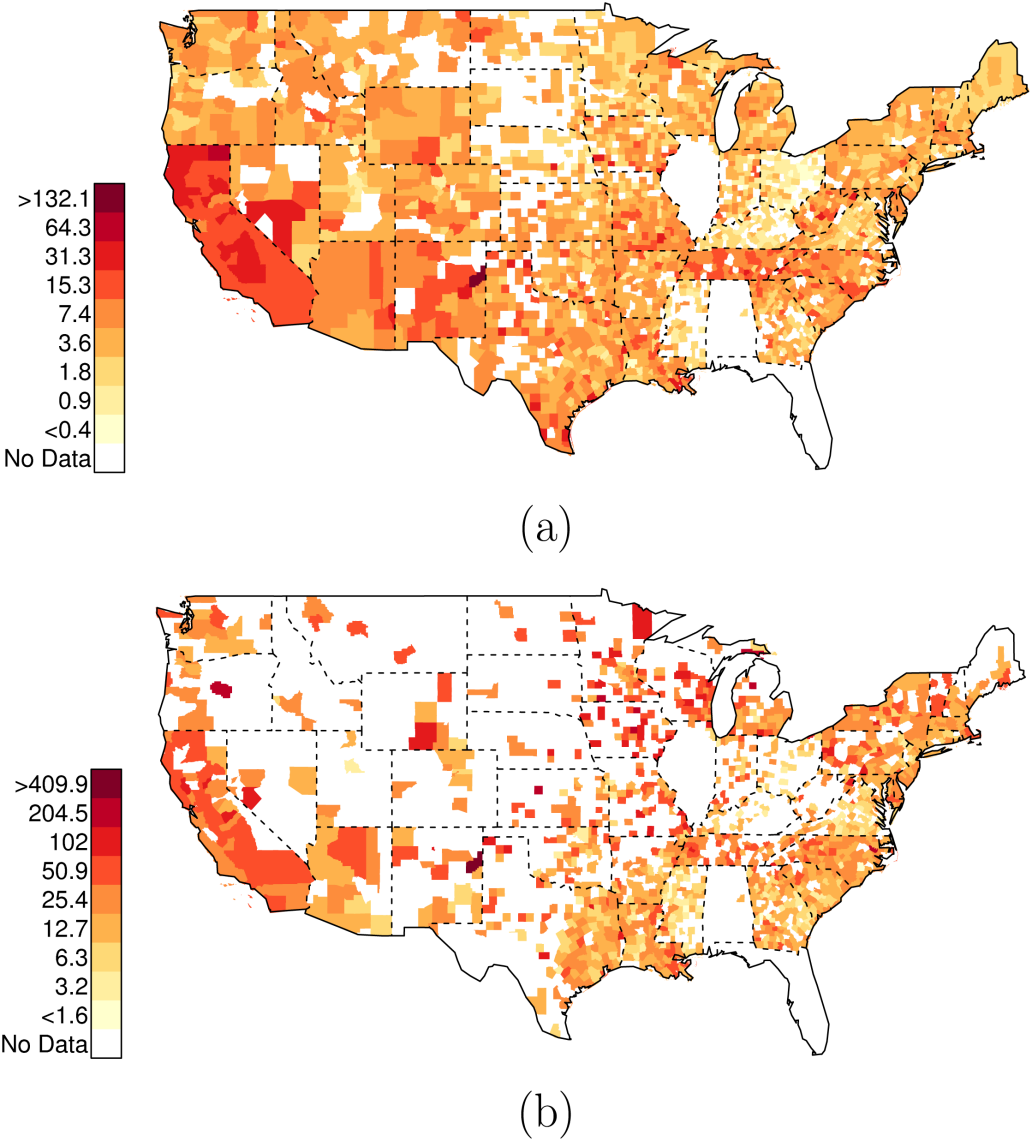
Data on Race-Specific Assault-Related Arrest Rates. In these figures, only counties with greater than one arrest are plotted. (a) County-specific Department of Justice data on assault-related arrests (White), per 10,000 residents (2012). (b) County-specific Department of Justice data on assault-related arrests (Black), per 10,000 residents (2012).

**Fig 11:**
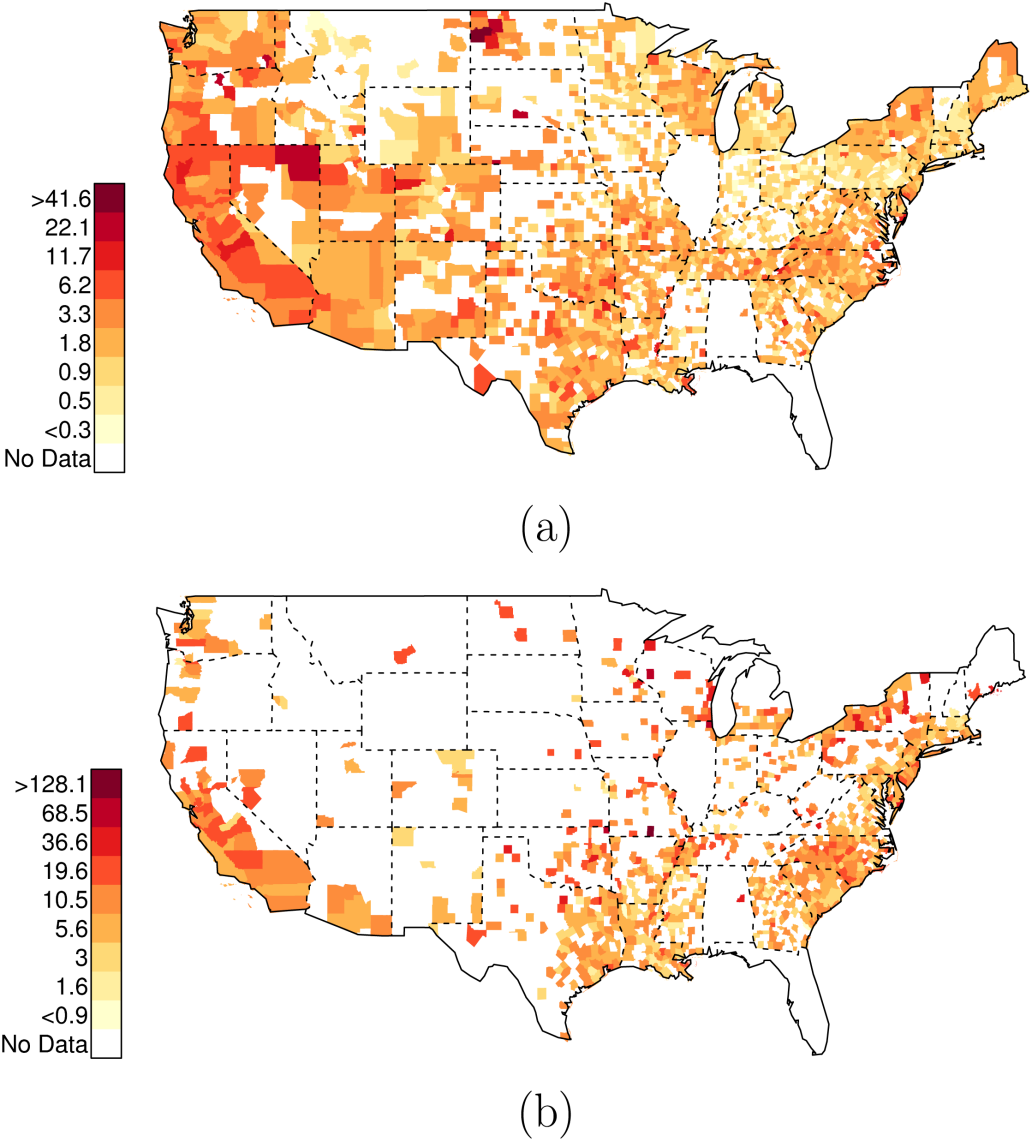
Data on Race-Specific Weapons-Related Arrest Rates. In these figures, only counties with greater than one arrest are plotted. (a) County-specific Department of Justice data on weapons-related arrests (White), per 10,000 residents (2012). (b) County-specific Department of Justice data on weapons-related arrests (Black), per 10,000 residents (2012).

**Fig 12:**
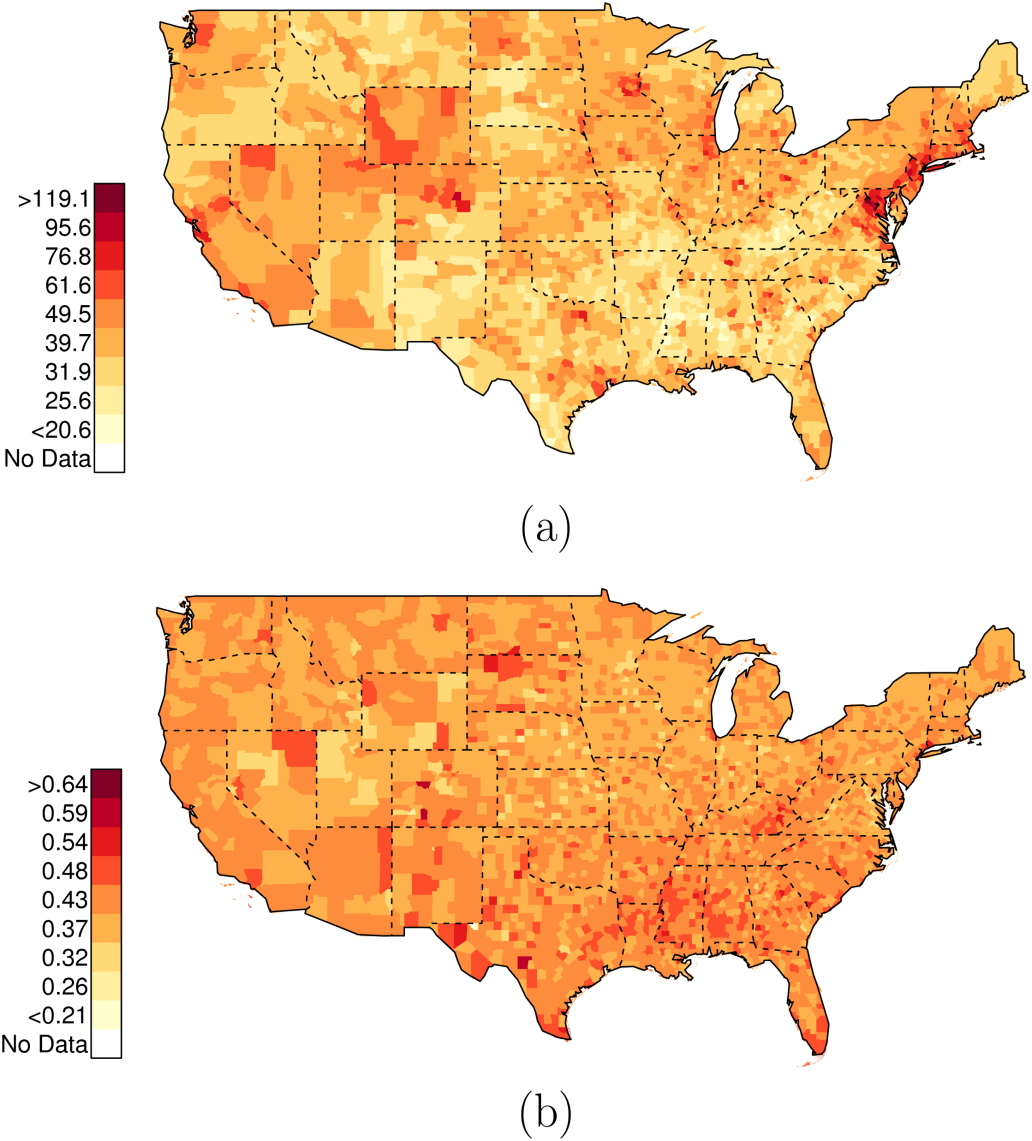
AIDSVu Data on Income and Inequality. (a) County-specific data on median income, in $1,000s. (b) County-specific data on inequality (Gini).

**Fig 13:**
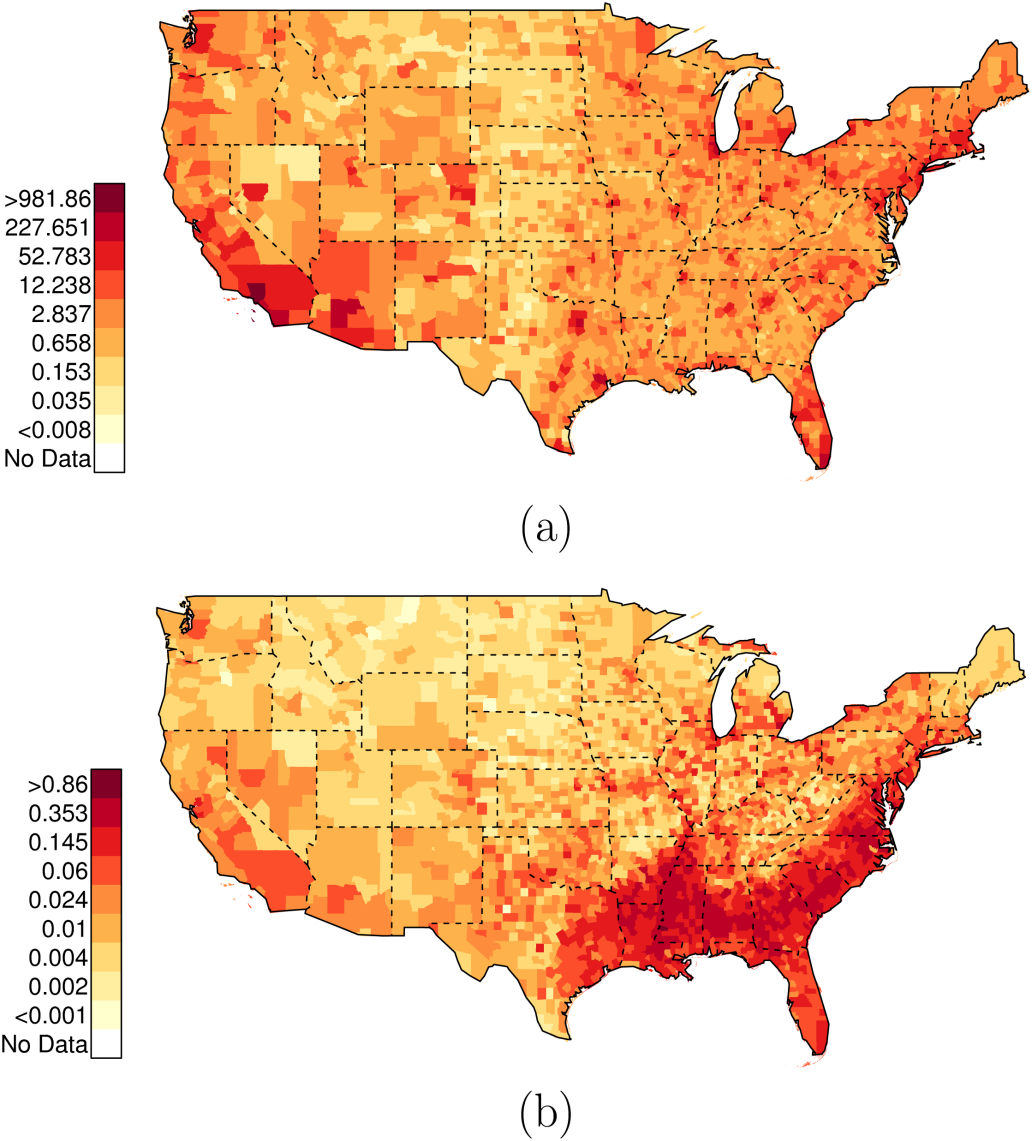
Population Data. (a) County-specific data on total population size, in 10,000s of residents. (b) County-specific data on the black-to-white population ratio.

**Fig 14:**
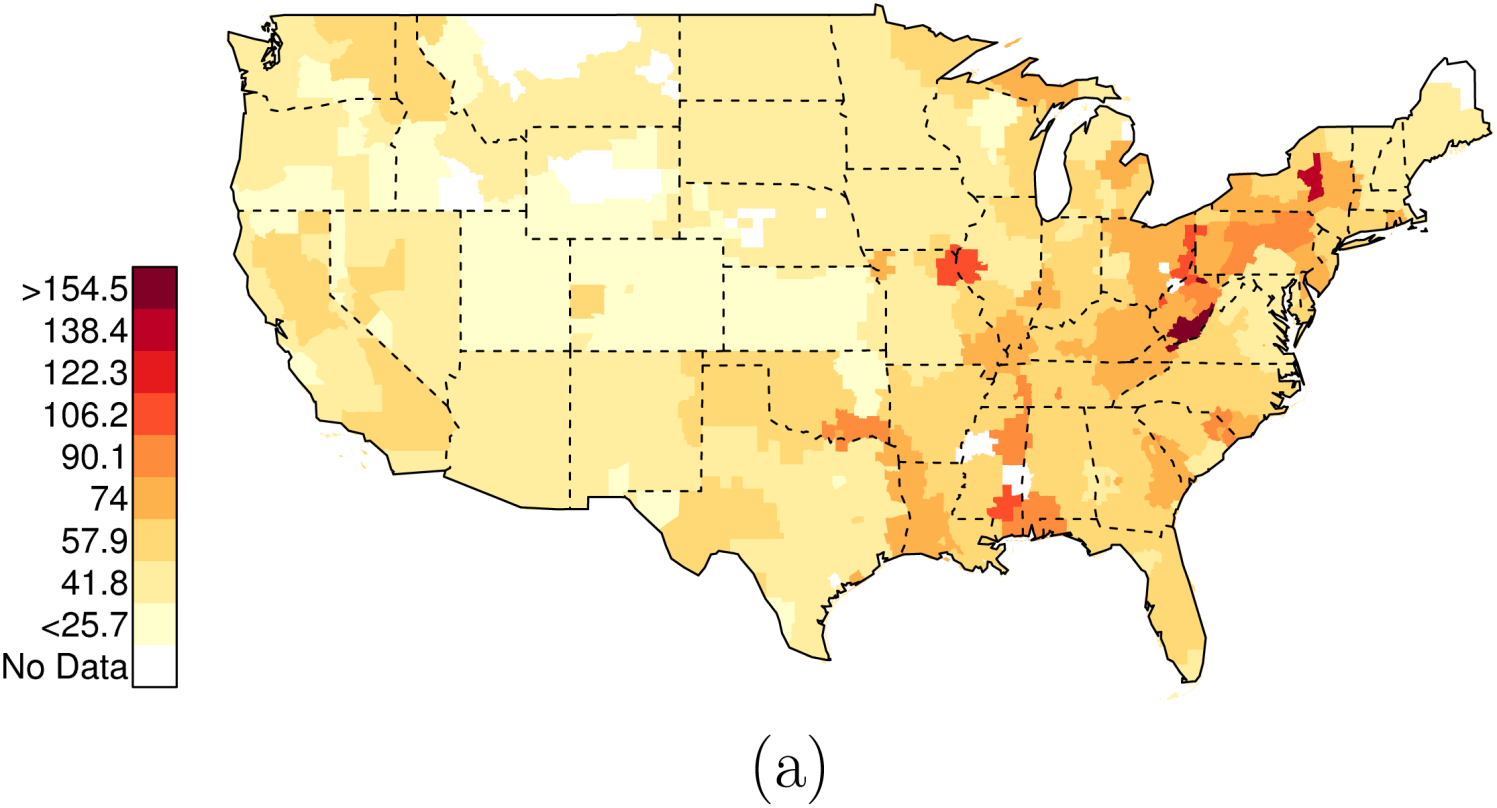
Data on Proxy Measure of Local Norms Concerning Racism. (a) Designated Market Area-specific Google Search Racism Scores, 2004–2007.

Figs 10, 11, 12, 13, and 14 present the geographic distributions of the predictor variables used in the analysis; the outcome variables are: 1) the risk ratio of {black, unarmed, and shot by police} to {white, unarmed, and shot by police}, and 2) the risk ratio of {black, unarmed, and shot by police} to {white, armed, and shot by police}.

Table 1 presents the results of modeling the risk ratio of {black, unarmed, and shot by police} to {white, unarmed, and shot by police}. Across models, there are some consistent trends: 1) population size is positively and reliably associated with the outcome, as is 2) the ratio of the black population size to the white population size; 3) median income shows a reliable negative association with the outcome; 4) the Gini index shows a reliable positive relationship with the outcome; 5) there is a consistently positive, though imprecisely estimated, relationship between the Google search data proxy of local-level racist norms and racial bias in police shooting; and, 6) there is no consistent relationship between the race-specific crime proxies (neither assault-related nor weapons-related arrest rates) and racial bias in police shootings.

**Table 1:** Predictors of an increased county-level risk of being {black, unarmed, and shot by police} relative to being {white, unarmed, and shot by police}. Values are: posterior mean (posterior standard deviation) of the regression coefficients. The symbol *log* referes to the natural logarithm. *Pop* refers to absolute population size. *Pct. B*. refers to the percentage of the county population that is black. *Md. In*. refers to median income. *Gini* refers to the Gini index of inequality. *GRP* refers to the Google search racism proxy. *W. Ast* and *B. Ast* refer to the white- and black-specific arrest rates for assualt, respectively. *W. Wps* and *B. Wps* refer to the white- and black-specific arrest rates for weapons violations, respectively. Posterior probabilty that a postive regression coeffcient is less than zero (or a negative one greater than zero) is coded as: * indicates a probability between 0.10 and 0.05, ** indicates a probability between 0.05 and 0.01, and *** indicates a probability of 0.01 or less.

Model	Intercept	SD	log(Pop.)	log(Pct. B.)	log(Md. In.)	log(Gini)	log(GRP)	log(W. Ast)	log(B. Ast)	log(W. Wps)	log(B. Wps)
M1	1.29 (0.06)	0.07 (0.04)									
M2	1.4 (0.08)	0.07 (0.04)	**0.1 (0.04)								
M3	1.55 (0.13)	0.06 (0.04)	*0.09 (0.06)	0.06 (0.05)							
M4	1.99 (0.4)	0.08 (0.04)	***0.12 (0.05)		*-0.42 (0.28)						
M5	2.4 (0.73)	0.07 (0.04)	0.06 (0.05)			*1.29 (0.94)					
M6	0.53 (1.04)	0.08 (0.04)	**0.1 (0.05)				0.21 (0.25)				
M7	1.41 (0.08)	0.06 (0.04)	**0.1 (0.05)					-0.02 (0.08)	-0.01 (0.09)		
M8	1.39 (0.09)	0.07 (0.03)	**0.1 (0.05)							0.03 (0.07)	0 (0.08)
M9	2.01 (0.21)	0.06 (0.03)	***0.11 (0.04)	**0.05 (0.03)	**-0.35 (0.16)						
M10	2.42 (0.68)	0.07 (0.04)	0.05 (0.05)	0.05 (0.06)		*1.21 (0.91)					
M11	0.79 (1.25)	0.1 (0.03)	**0.08 (0.05)	0.05 (0.06)			0.18 (0.29)				
M12	1.5 (0.13)	0.11 (0.03)	0.06 (0.06)	0.06 (0.05)				-0.01 (0.08)	0.03 (0.09)		
M13	1.51 (0.14)	0.08 (0.03)	*0.07 (0.05)	0.06 (0.06)						0.02 (0.09)	0.01 (0.08)
M14	2.32 (0.61)	0.06 (0.04)	0.08 (0.07)	0.05 (0.06)	-0.27 (0.34)	0.58 (1.01)					
M15	1.8 (1.38)	0.12 (0.03)	**0.1 (0.06)	0.04 (0.07)	-0.38 (0.3)		0.05 (0.29)				
M16	2.02 (0.44)	0.06 (0.04)	**0.1 (0.06)	0.05 (0.05)	-0.37 (0.3)			-0.03 (0.08)	-0.01 (0.08)		
M17	2.07 (0.36)	0.09 (0.03)	*0.08 (0.05)	0.06 (0.05)	**-0.43 (0.26)					0 (0.07)	0.01 (0.08)
M18	1.83 (1.39)	0.08 (0.03)	0.07 (0.06)	0.04 (0.06)		0.99 (0.87)	0.1 (0.28)				
M19	2.26 (0.69)	0.06 (0.04)	0.06 (0.05)	0.04 (0.06)		1 (0.92)		-0.01 (0.09)	0.01 (0.08)		
M20	2.28 (0.67)	0.08 (0.03)	0.05 (0.05)	0.05 (0.06)		1.01 (0.89)				0.01 (0.09)	0 (0.08)
M21	1.39 (1.33)	0.1 (0.03)	**0.1 (0.06)	0.05 (0.06)	-0.33 (0.28)		0.14 (0.29)	-0.02 (0.08)	0.01 (0.07)		
M22	1.59 (1.31)	0.07 (0.03)	**0.1 (0.05)	0.04 (0.06)	-0.36 (0.31)		0.1 (0.28)			0.01 (0.08)	-0.02 (0.08)
M23	1.83 (1.42)	0.11 (0.03)	0.06 (0.05)	0.03 (0.06)		0.98 (0.89)	0.1 (0.28)	-0.02 (0.08)	0.01 (0.08)		
M24	1.68 (1.45)	0.07 (0.04)	0.06 (0.05)	0.03 (0.07)		0.97 (0.86)	0.13 (0.3)			0.02 (0.09)	-0.01 (0.08)
M25	1.95 (1.45)	0.08 (0.04)	*0.09 (0.06)	0.03 (0.07)	-0.27 (0.34)	0.64 (0.99)	0.09 (0.29)	-0.03 (0.09)	0 (0.09)	0.03 (0.1)	-0.02 (0.09)

Table 2 presents the results of modeling the risk ratio of {black, unarmed, and shot by police} to {white, armed, and shot by police}. In this case, there are much more reliable positive effects for: 1) population size, and 2) the ratio of black population size to the white population size. As before, 3) median income shows a negative association with the outcome, and 4) the Gini index shows a positive relationship with the outcome; 5) there is a consistently positive, though imprecisely estimated, relationship between the Google search data proxy of local-level racist norms and racial bias in police shooting; and, 6) there is no consistent relationship between the race-specific crime proxies (neither assault-related nor weapons-related arrest rates) and racial bias in police shootings.

**Table 2:** Predictors of an increased county-level risk of being {black, unarmed, and shot by police} relative to being {white, armed, and shot by police}. Values are: posterior mean (posterior standard deviation) of the regression coefficients. The symbol *log* referes to the natural logarithm. *Pop* refers to absolute population size. *Pct. B*. refers to the percentage of the county population that is black. *Md. In*. refers to median income. *Gini* refers to the Gini index of inequality. *GRP* refers to the Google search racism proxy. *W. Ast* and *B. Ast* refer to the white- and black-specific arrest rates for assualt, respectively. *W. Wps* and *B. Wps* refer to the white- and black-specific arrest rates for weapons violations, respectively. Posterior probabilty that a postive regression coeffcient is less than zero (or a negative one greater than zero) is coded as: * indicates a probability between 0.10 and 0.05, ** indicates a probability between 0.05 and 0.01, and *** indicates a probability of 0.01 or less.

Model	Intercept	SD	log(Pop.)	log(Pct. B.)	log(Md. In.)	log(Gini)	log(GRP)	log(W. Ast)	log(B. Ast)	log(W. Wps)	log(B. Wps)
M1	0.15 (0.07)	0.11 (0.05)									
M2	0.4 (0.08)	0.09 (0.04)	***0.25 (0.05)								
M3	0.64 (0.15)	0.08 (0.05)	***0.21 (0.05)	**0.13 (0.06)							
M4	1.18 (0.43)	0.07 (0.05)	***0.29 (0.05)		**-0.57 (0.3)						
M5	1.78 (0.73)	0.1 (0.04)	***0.21 (0.05)			**1.77 (0.93)					
M6	-0.84 (1.15)	0.09 (0.04)	***0.25 (0.05)				0.3 (0.28)				
M7	0.41 (0.08)	0.07 (0.05)	***0.25 (0.05)					-0.07 (0.1)	-0.01 (0.1)		
M8	0.38 (0.1)	0.08 (0.05)	***0.25 (0.05)							0.01 (0.11)	0.03 (0.09)
M9	1.17 (0.44)	0.09 (0.05)	***0.24 (0.06)	**0.11 (0.06)	-0.39 (0.32)						
M10	1.6 (0.72)	0.08 (0.04)	***0.19 (0.06)	*0.1 (0.06)		*1.29 (0.94)					
M11	0.09 (1.26)	0.08 (0.04)	***0.21 (0.05)	*0.11 (0.07)			0.13 (0.29)				
M12	0.65 (0.15)	0.09 (0.04)	***0.21 (0.05)	**0.12 (0.06)				-0.06 (0.09)	0.02 (0.1)		
M13	0.62 (0.14)	0.08 (0.05)	***0.2 (0.05)	**0.12 (0.06)						0 (0.1)	0.04 (0.09)
M14	1.69 (0.69)	0.08 (0.04)	***0.21 (0.06)	*0.1 (0.07)	-0.3 (0.35)	0.88 (1.04)					
M15	0.93 (1.41)	0.09 (0.05)	***0.24 (0.06)	*0.1 (0.07)	-0.39 (0.33)		0.06 (0.3)				
M16	1.29 (0.45)	0.09 (0.04)	***0.26 (0.07)	*0.09 (0.07)	*-0.49 (0.32)			-0.07 (0.1)	0 (0.09)		
M17	1.22 (0.47)	0.08 (0.04)	***0.24 (0.06)	**0.11 (0.06)	*-0.43 (0.33)					-0.02 (0.1)	0.02 (0.09)
M18	1.54 (1.56)	0.11 (0.04)	***0.18 (0.06)	*0.09 (0.07)		*1.4 (0.96)	0.03 (0.31)				
M19	1.6 (0.75)	0.08 (0.04)	***0.19 (0.06)	*0.09 (0.07)		*1.29 (0.99)		-0.06 (0.09)	0 (0.1)		
M20	1.62 (0.72)	0.12 (0.04)	***0.18 (0.05)	*0.1 (0.07)		*1.33 (0.95)				0 (0.1)	0.03 (0.09)
M21	0.97 (1.46)	0.09 (0.05)	***0.25 (0.06)	*0.09 (0.07)	*-0.46 (0.33)		0.07 (0.31)	-0.06 (0.09)	-0.02 (0.1)		
M22	0.96 (1.54)	0.11 (0.04)	***0.24 (0.06)	*0.11 (0.07)	-0.41 (0.33)		0.06 (0.33)			-0.01 (0.1)	0.01 (0.09)
M23	1.39 (1.54)	0.12 (0.03)	***0.19 (0.06)	*0.09 (0.07)		1.2 (0.98)	0.04 (0.31)	-0.06 (0.1)	0.01 (0.1)		
M24	1.35 (1.55)	0.1 (0.04)	***0.19 (0.06)	*0.1 (0.07)		*1.24 (0.93)	0.05 (0.31)			0.01 (0.1)	0.03 (0.09)
M25	1.55 (1.58)	0.08 (0.04)	***0.22 (0.06)	0.08 (0.07)	-0.3 (0.38)	0.86 (1.08)	0.03 (0.31)	-0.07 (0.11)	-0.02 (0.11)	0.03 (0.1)	0.02 (0.1)

In effect, larger county population size, a higher proportion of black residents in the population, lower median income, and greater disparities in income all appear to be reliably associated with an elevated ratio of police shooting rate against unarmed black individuals relative to unarmed—and even armed—whites.

In each model that considers them, race-specific crime rates are always entered as simultaneous predictors (see Tables 1 and 2). This model parameterization allows us to examine the effects of race-specific crime rates on racial bias in police shootings. However, there are questions that this model parameterization precludes. Most importantly, having an aggregated measure of crime rate would allow one to test the questions: 1) does racial bias in police shooting increase in areas where crime is generally more prevalent? And, 2) as the difference of black crime rate minus white crime rate increases, does racial bias in police shootings also increase?

As a robustness check, the results from two alternative model parameterizations in predicting the relative risk of being {unarmed, black, and shot by police} to being {unarmed, white, and shot by police} are presented. These models are based on including the sum and difference of race-specific crime rates in the regression; see *Appendix.pdf* in S1 File. The results of these supplementary models are qualitatively the same as those of the main models; racial bias in police shooting is not reliably associated with crime rate and not related to the difference in race-specific crime rates.

## Discussion

It is important to reiterate that these risk ratios come only from the sample of individuals who were shot by police and census data on race/ethnicity-specific population information. The USPSD does not have information on encounter rates between police and subjects according to ethnicity. As such, the data cannot speak to the relative risk of being shot by a police officer conditional on being encountered by police, and do not give us a direct window into the psychology of the officers who are pulling the triggers. The racial biases and behaviors of officers upon encountering a suspect could clearly be components of the relative risk effects observed in the data, but other social factors could also contribute to the observed patterns in the data. More specifically, heterogeneity in encounter rates between suspects and police as a function of race could play a strong role in the racial biases in shooting rates presented here.

### Shot by Police: Armed Versus Unarmed

At face value, the data suggest that conditional on being of a given race/ethnicity and being shot by police, one was more-likely-than-not to be armed. It is interesting to note that the armed-to-unarmed risk ratio in whites is elevated relative to that of black or hispanic individuals (especially in a handful of outlier counties, see Data folder in S1 File). This pattern is consistent with police being more discerning of armed/unarmed status before shooting a white suspect than shooting a black or hispanic suspect.

At a broader level, conditional on being shot by police, the probability ratio of being armed rather than unarmed averages around 3 to 1, with variation depending on race/ethnicity and location. While it is true that vast majority of police contacts are presumably with unarmed people, and that police shoot only a small fraction of the unarmed people they encounter, the public, and even some police chiefs, still lament that the use of deadly force is probably avoidable in a non-negligible portion of police shootings, if for no other reason than the fact that the suspects/civilians are unarmed.

Chief of Police Chris Magnus of the Richmond Police Department in California, for example, details many ways in which use of force by police officers can be attenuated, while increasing the safety of both the officers and public [40]. Magnus argues that police often do not come from or live in the communities they police, and placement is not necessarily random—police can request the kinds of environments they prefer, even if such placements are not beneficial to the community; for example, underlying racial biases could lead certain kinds of white police officers to request work in predominately black neighborhoods. Magnus suggests that a way around creating conflicts of interest between the community and the police involves thinking:

“… about what kind of folks you want to attract to your department, and how you do that. You look at some departments’ recruiting materials, and you see guys jumping out of trucks in SWAT gear and people armed with every imaginable weapon… My goal, at least, is to look for people who want to work in my community, not because it’s a place where they think they’re going to be dealing with a lot of violence and hot chases and armed individuals and excitement and an episode of Cops or something… I want them to be here because they’re interested in building a partnership with the community” [40].

Following Magnus’ line of logic, one might expect that some of the racial bias in police killings, as well as the rate of shooting unarmed civilians, could be attenuated by requiring police departments to adopt hiring and policing practices like those being used successfully in Richmond, where no civilians (armed or unarmed) were killed by police for more than five years in spite of Richmond having a large population size (107,000) [40].

### Shot by Police: Across Race/Ethnicity

Across almost all counties, individuals who were armed and shot by police had a much higher probability of being black or hispanic than being white. Likewise, across almost all counties, individuals who were unarmed and shot by police had a much higher probability of being black or hispanic than being white. Tragically, across a large proportion of counties, individuals who were shot by police had a higher median probability of being *unarmed* black individuals than being *armed* white individuals. While this pattern could be explained by reduced levels of crime being committed by armed white individuals, it still raises a question as to why there exists such a high rate of police shooting of unarmed black individuals.

The posterior estimates of the relative risk ratios as a function race and armed/unarmed status are included for all counties in the Data folder of S1 File. From this data, one can identify the counties and police departments where racial bias in shooting rates is strongest. Counties like Miami-Dade (FL), Cook (IL), Los Angeles (CA), Orleans Parish (LA), Pulaski (AR), Harris (TX), Baltimore (MD), and Allegheny (PA), stand out as departments where police protocols may benefit from review.

### County-Level Predictors of Racial Bias in Police Shooting

In predicting the risk ratio of {black, unarmed, and shot by police} to {white, unarmed, and shot by police}, there are effects of county-level population size, racial composition, median income, and Gini score. In predicting the risk ratio of {black, unarmed, and shot by police} to {white, armed, and shot by police}, there are more reliable effects for these same variables. These findings suggest that racial bias in police shootings is most common among police working in larger metropolitan counties with low median incomes and a sizable portion of black residents, especially when there is high financial inequality in that county. These results are consistent with much of the previous work outlined in the introduction.

It is sometimes suggested that in urban areas with more black residents and higher levels of inequality, individuals may be more likely to commit violent crime, and thus the racial bias in police shooting may be explainable as a proximate response by police to areas of high violence and crime (community violence theory [14, 15, 23, 35]). In other words, if the environment is such that race and crime covary, police shooting ratios may show signs of racial bias, even if it is crime, not race, that is the causal driver of police shootings. In the models fit in this study, however, there is no evidence of an association between black-specific crime rates (neither in assault-related arrests nor in weapons-related arrests) and racial bias in police shootings, irrespective of whether or not other controls were included in the model. As such, the results of this study provide no empirical support for the idea that racial bias in police shootings (in the time period, 2011–2014, described in this study) is driven by race-specific crime rates (at least as measured by the proxies of assault- and weapons-related arrest rates in 2012).

The methodology used in this paper does not allow one to speak with confidence as to the causal drivers of racial bias in police shooting; the source of racial bias in police shootings, however, can be logically decomposed into two parts: 1) racial bias in encounter rates between police and suspects/civilians, and/or 2) racial bias in use of force upon encountering these suspects/civilians. A racial bias in encounter rates could be unjustifiable (police engage in racist or ethnic targeting of blacks/hispanics irrespective of suspected criminal activity) or a proportional response to local-level, race/ethnicity-specific, crime rates. Under the assumptions that police express no racial bias in use of force upon encountering suspects/civilians, and also engage in interactions with suspects/civilians in direct proportion to race/ethnicity-specific crime rates (where crime rates covary with race/ethnicity), one would expect to see an association between racial bias in police shootings and race-specific crime rates—an association that is not found in these results. As such, the results of this study provide evidence that there is racial bias in police shootings that is not explainable as a response to local-level crime rates, and is related to either: 1) racial bias in police encountering suspects/civilians, or 2) racial bias by police in the use of force upon encountering suspects/civilians.

The geographically-resolved proxy of racial animus used in this study, however, did not show a reliable association with racial bias in police shootings, although the association was consistently positive across models. This finding does not rule out the possibility that racist norms within police departments themselves may potentially have much stronger associations with racial bias in police shootings than these more coarse ecological-level data. Many police, or former police, report of—or have been documented to engage in promotion of—extensive racist norms (e.g. see [41–49], and note that this list is far from exhaustive). Acquiring more systematic data on the extent of racist norms within police departments (as opposed to the counties in which they are clustered) will require more thorough qualitative and quantitative investigations of police departments themselves.

### Conclusions and Future Directions

#### Completing the Police Shootings Database

While the USPSD already contains almost 2,000 records of police shootings, as many as 55% of the days to be screened for police shootings have yet to be scoured for incidents. While the currently available data are sufficient to investigate risk ratios across racial/ethnic categories, estimation of the absolute risk of being shot by police requires the completed data set. Furthermore, the completed data and absolute risk estimates will help to identify the departments and officers with disproportionately high: 1) absolute shooting rates, 2) rates of shooting unarmed civilians, and 3) rates of racially-biased shootings. Although an increased completeness of the data is unlikely to change the estimated mean effects presented in this analysis, since the sample used herein is a large and random subset of the to-be-completed data set, more data will allow for more precise estimates at the county-level, and may expand the number of counties that can be modeled at the ecological-level.

After data collection is complete, all new entries still require verification and archival of the primary sources of information. As with the data collection process until now, this phase of the project will require assistance from the public.

As new data are fact-checked and verified, it might be possible to expand the scope of variables included in the database. For instance, a qualitative assessment could be provided, indicating whether the shooting was clearly justified (for example, a lethal police response was immediately necessary and unavoidable to protect the lives of innocents), clearly unjustified (for example, a suspect was shot in the back while restrained, as was the case in the execution of Oscar Grant III [50]), or somewhere in between. To mitigate bias, the coding could be conducted by several individuals, and the suite of all responses could be used to construct confidence in the assessment.

Likewise, data could be classified on the basis of more direct empirical evidence. For example: was the suspect shot in the back? Was the suspect restrained when shot? Also, what were the circumstances surrounding the shooting? Were police called on report of a serious crime? Was the situation escalated by police prior to shooting? Does the race/ethnicity of a suspect vary by the circumstance surrounding the shooting? What was the race/ethnicity of the shooter? Are police more likely to escalate a situation when the suspect is black?

#### More Detailed Analysis of the Data

More detailed analysis of the data is needed. In the current analysis, county-specific risk ratios are estimated. In the future, these estimates could be extended in a Bayesian framework to include estimates unique to police departments, as clustered into counties, as clustered into states.

The analyses presented here are only a very rough first pass through the data. As the data become more complete, more nuance in analysis is needed. The estimated levels of unjustifiable racial bias in police shootings will likely change when reliable estimates of justification are included in the analysis, and when spatial correlations and ecological covariates are formally included in the model being used to estimate risk ratios.

In this paper, the relationship between race/ethnicity and status of a civilian/suspect as armed or unarmed on the relative probability of being shot by police is investigated, but there are differences between the categories of armed/unarmed and justified/unjustified, which should be taken into account in future research.

#### Higher Quality Covariate Data is Needed

Ecological regression on county-level characteristics is plagued by difficulties theoretically [39, 51]; issues with data quality make it even harder to use county-level data. In the analysis of county-level predictors of racial bias in police shootings conducted in this paper, some of the data were low quality. Notably, the crime data may be biased by the reporting practices of the police, and Florida, Alabama, and Illinois failed to fully release data, which led to the use Bayesian imputation for counties in these states.

#### Towards Attenuation of Racial Bias in Police Violence

Hopefully, a more transparent representation of police homicide and shooting data as a function of: 1) the race/ethnicity of suspects/civilians, 2) the status of suspects/civilians as armed/unarmed, and 3) the geographic location of the incidents will help the public, academic researchers, and federal agencies to better evaluate the practices of police in the United States. In addition to assisting in the testing hypotheses related to the structural drivers of racial bias in police homicide across the nation, these data can be used to justify more in-depth qualitative investigations by academics, journalists, and watchdog organizations of the subset of police jurisdictions with the most racially-biased police shooting rates.

Such in-depth investigations may help to identify the locally contextualized causes of racial bias in police homicide. Critically important in evaluating attenuation (or lack thereof) of racial bias in police violence are time series data on the extent of racial bias at the local level; hopefully, continued enthusiasm for citizen driven data collection will provide researchers with the data needed to study and publish results documenting temporal trends in racial bias in police shootings.

Much traditional academic work on the topic of police violence has focused on comparison of multiple theoretical causes as listed earlier. However, it is likely that all of the above-listed causes contribute to police shootings, but that the relative weight of each potential driver of racialized police violence may be heterogeneous over geography and time [52, 53]. As such, it may be valuable to transition from focusing on questions like: “What explains the racial bias in police shootings?” to questions like: “What explains the racial bias of police shootings in Miami-Dade county in 2011–2014?”.

Attenuating the racial bias in police shootings in a given location requires a better understanding of the drivers of racial bias operating in that location. Databases like the USPSD cannot directly provide answers to such questions, but they can help identify where more detailed ethnographic investigations should be conducted. Perhaps police departments with disproportionate rates of racially-biased police homicide can provide justification for these patterns based on local context, or perhaps they are headed by individuals like Police Commander Jon Burge—the public needs to know which.

## Methods

### Data

#### U.S. Police Shootings Data

Data on police shootings were accessed in December 2014, from the U.S. Police Shootings Database [1] compiled by Kyle Wagner. The data can be accessed and improved at http://goo.gl/Su60Cm.

From the total set of police shooting data, records were selected only when: 1) a suspect’s status as either armed or unarmed was clearly described (sometimes by searching for subsequent media reports), 2) a suspect’s race/ethnicity could be clearly assigned, 3) the year of the shooting was no earlier than 2011 and no later than 2014, and 4) a county name could be assigned. The data were then error-checked by removing listings for the same suspect/victim name; duplicates under spelling variations of suspect/victim name were found and eliminated by checking for multiple cases of police shootings with the same victim year, age, race/ethnicity, and county. In total, 721 cases of police shootings were analyzed. About five percent (N = 35) of records were randomly spot-checked by using a random number generator to sort the cleaned Excel file containing the data; the urls cited in the USPSD were then checked to ensure validity of the data. In each case the urls were valid (but see below) and the data accurately described. In a small number of cases (N = 3) the original url returned an error and www.archive.org was used to retrieve the original web-page, which, in each case, was archived under the url provided in the USPSD and included a full event description. The raw data of the USPSD in its entirety can be found at http://goo.gl/QrylkY. Additionally, the cleaned data modeled in this paper are included in the the Data folder of S1 File. Finally, county-level raw data, covariates, and modeling results are included in the Data folder of S1 File in a geographically resolved format linked to a shapefile that can be used for geographic data visualization in the R software environment.

Methodologically, users are asked to adhere to the following protocol in data collection (see [1], direct quotation follows):

1. Using Google’s search tools, isolate a single day (e.g. Jan. 1, 2011, to Jan. 1, 2011) and search for the term “police involved shooting” (don’t use quotation marks). Use Chrome’s Incognito mode when searching to ensure you aren’t getting local results.
2. Read each link on the first 10 pages of results; for any instances of shootings involving a police officer, log them in the form.
3. We’re looking at 2011, 2012, [2013 and 2014], and tracking date, name, age, gender, race/ethnicity, injured/killed, and a number of other fields. Please be as thorough as possible with each incident, and provide links to where you found the information (this will be crucial during verification). Often, the first day of reports will not have personal details, and a second search of subsequent days will fill in more of the story.
4. Before starting in, take a look at the submissions here and pick a day that no one has begun (“Not Checked” in the third sheet). Remember, we’re starting off by looking at just the past three years.
5. A later death, after a person is hospitalized in a police-involved shooting, is considered a death for our purposes.
6. We are looking for any incidence of a police officer shooting and hitting another person. This can be off-duty if the officer was acting in a law-enforcement capacity.
7. We are not looking for incidences of police officers discharging their weapons and hitting no one. In a perfect world these would be tracked, since often the only difference is that the shot missed, but these incidents are not as thoroughly reported and would probably bias the data.
8. Please keep the data as neat as possible. Work within specific months, make sure you’re in the correct year, keep the columns clean, add peripheral information in the “Summary” portion, etc.

#### Census and Covariate Data

County-level census population data and county-level covariates on income and inequality were accessed from AIDSvu [54], an open access database available through the Rollins School of Public Health at Emory University.

#### Google Racial Animus Data

The racial animus data were generously provided by Seth Stephens-Davidowitz [29] at the level of the DMA (designated market area), and were translated to county-level data using FIPS codes and a cross-walk provided by the ICPSR at http://www.icpsr.umich.edu/icpsrweb/DSDR/studies/22720.

#### Crime Rate Data

Race-specific assault- and weapons-related arrest data compiled by the United States Department of Justice were accessed from the Inter-University Consortium for Political and Social Research [55]. Arrests were summed within counties.

#### Geographic Data

Geographic shapefiles were downloaded from GADM [56], an open access database of Global Administrative Areas.

### Bayesian Modeling

#### Estimating Risk Ratios for Police Shooting as a Function of Race and Arms Status

County-level police shooting rates are estimated using binomial probabilities, and a prior, estimated from the data, under hierarchical partial pooling. Hierarchical pooling allows information collected in other counties within the United States to partially inform the parameter estimates in a focal county, which improves out-of-sample predictive inference globally [57], and regularizes otherwise undefined posterior predictive risk ratios. Prior to the introduction of multi-level modeling methods, relative risk ratios at local levels were very hard to infer. For example, if no unarmed whites but three unarmed black individuals were killed in a county, the estimated relative risk ratio would be:

$$\begin{array}{c} \frac{\frac{3}{N_{\left[c\right]}^{B}}}{\frac{0}{N_{\left[c\right]}^{W}}}=\frac{\text{constant}}{0} \end{array}$$

yielding some undefined relative risk ratio. The multi-level Bayesian methods used here, partially (rather than fully) pool information across counties, allowing for more stable estimates in relative risk ratios (by regularizing the denominator of the above equation away from zero and toward the global mean for that value), while still allowing for, and representing, heterogeneity across counties.

To begin the model, the race-specific probabilities of being shot by police at the county-level are estimated under two conditions (armed and unarmed), in all counties with at least a single incidence of a police-involved shooting between the years of 2011–2014. These probabilities are then used to estimate the relative risk of being the victim of a police shooting, conditional on being armed or unarmed, and race/ethnicity.

For example, the probability, $\theta_{\left[c\right]}^{B,U}$, in county *c* of a black (B) and unarmed (U) individual being shot by police can be estimated from the county-level census data, $N_{\left[c\right]}^{B}$, and the count of shootings, $S_{\left[c\right]}^{B,U}$, as:

$$\begin{array}{c} S_{\left[c\right]}^{B,U}∼\text{Binomial}\left(N_{\left[c\right]}^{B},\theta_{\left[c\right]}^{B,U}\right) \end{array}$$ (1)

Eq (1) serves as a template for each of the main probability statements in the model, where a probability unique to the interaction of race/ethnicity (B = black, H = hispanic, and W = white) and arms status (A = armed and U = unarmed) is estimated. Note that superscripts are labels, and do not indicate exponentiation. The subsequent main probability statements run as follows:

$$\begin{array}{c} S_{\left[c\right]}^{B,A}∼\text{Binomial}\left(N_{\left[c\right]}^{B},\theta_{\left[c\right]}^{B,A}\right) \end{array}$$ (2)

$$\begin{array}{c} S_{\left[c\right]}^{H,U}∼\text{Binomial}\left(N_{\left[c\right]}^{H},\theta_{\left[c\right]}^{H,U}\right) \end{array}$$ (3)

$$\begin{array}{c} S_{\left[c\right]}^{H,A}∼\text{Binomial}\left(N_{\left[c\right]}^{H},\theta_{\left[c\right]}^{H,A}\right) \end{array}$$ (4)

$$\begin{array}{c} S_{\left[c\right]}^{W,U}∼\text{Binomial}\left(N_{\left[c\right]}^{W},\theta_{\left[c\right]}^{W,U}\right) \end{array}$$ (5)

$$\begin{array}{c} S_{\left[c\right]}^{W,A}∼\text{Binomial}\left(N_{\left[c\right]}^{W},\theta_{\left[c\right]}^{W,A}\right) \end{array}$$ (6)

Thus, in each county, there are D = 6 probability parameters to be estimated. These parameters are organized into a vector:

$$\begin{array}{c} \theta_{\left[c\right]}={\left(\theta_{\left[c\right]}^{B,U},\theta_{\left[c\right]}^{B,A},\theta_{\left[c\right]}^{H,U},\theta_{\left[c\right]}^{H,A},\theta_{\left[c\right]}^{W,U},\theta_{\left[c\right]}^{W,A}\right)}^{\prime } \end{array}$$ (7)

The probability parameter vector *θ*_[*c*]_ ∈ (0,1)^*D*^ is transformed to a parameter vector on the unconstrained scale Θ_[*c*]_ ∈ ℝ^*D*^ using an inverse logit transformation:

$$\begin{array}{c} \theta_{\left[c\right]}=\frac{e^{\Theta_{\left[c\right]}}}{1+e^{\Theta_{\left[c\right]}}} \end{array}$$ (8)

Then, vectors unique to each county *c*, are estimated from a higher-level distribution in a multi-level Bayesian framework:

$$\begin{array}{c} \Theta_{\left[c\right]}∼\text{Multivariate}\;\text{Normal}\;\text{Cholesky}\left(\mu ,L\right) \end{array}$$ (9)

The Cholesky factor, *L*, of the model’s covariance matrix is formed from Cholesky decomposition of the correlation matrix *ρ* multiplied by a diagonal matrix of scale parameters, *σ*:

$$\begin{array}{c} L=\text{Diag}(\sigma )\times \text{Cholesky}\;\text{Decompose}(\rho ) \end{array}$$ (10)

Each element of the mean vector, *μ*, is given a weakly regularizing normal prior:

$$\begin{array}{c} \mu ∼\text{Normal}(-14,4) \end{array}$$ (11)

which places weakly regularizing prior support over the plausible range of values on the probability scale. Each element of the scale vector, *σ*, is given a vague half-Cauchy prior:

$$\begin{array}{c} \sigma ∼\text{Cauchy}(0,5)T[0,\infty ] \end{array}$$ (12)

The correlation matrix is given an implicit uniform prior over its support. The parameters of the model are estimated using Hamiltonian Markov Chain Monte Carlo simulation, implemented in the Stan version 2.5 C++ library [58].

The raw probability estimates at the county-level are surely underestimates, because the U.S. Police Shootings Database has still only documented a fraction of the police shootings since 2011. However, since the data are collected by users selecting a given day, and then recording information on police shootings documented on that day, the data are randomly collected with respect to the suspect’s race/ethnicity, location, and status as armed versus unarmed. As such, the relative risk ratios of U.S. citizens to police shooting as a function of race/ethnicity, location, or armed status should not be especially biased. Relative risk ratios are notated as below:

$$\begin{array}{c} {R_{\left[c\right]}}^{\begin{array}{c} B,U\\ W,U \end{array}}=\frac{\theta_{\left[c\right]}^{B,U}}{\theta_{\left[c\right]}^{W,U}} \end{array}$$ (13)

The symbol ${R_{\left[c\right]}}^{\begin{array}{c} B,U\\ W,U \end{array}}$ reads as: “The relative risk in county *c* of being black, unarmed, and shot by police, to being white, unarmed, and shot by police.”

#### Modeling Risk Ratios for Police Shooting as a Function of County-Level Covariates

In each county, the relevant risk ratio, *R*_[*c*]_, is modeled as a function of county-level covariates. To begin the model, each posterior sample of *R*_[*c*]_ from the above described Bayesian model is log-transformed and the distribution is summarized by calculating the mean, *μ*_*R*_[*c*]__ = mean(log(*R*_[*c*]_)), and standard deviation, *σ*_*R*_[*c*]__ = sd(log(*R*_[*c*]_)) over the MCMC samples. Uncertainty about these estimates is propagated using a Bayesian measurement error model:

$$\begin{array}{c} Y_{\left[c\right]}∼\text{Normal}\left(\mu_{R_{\left[c\right]}},\sigma_{R_{\left[c\right]}}\right) \end{array}$$ (14)

$$\begin{array}{c} Y_{\left[c\right]}∼\text{Normal}\left(\mu_{\left[c\right]},\sigma \right) \end{array}$$ (15)

where *μ*_[*c*]_ is defined for several candidate models (M1, M2, … M25), using a standard regression equation. The most basic, intercept only, model is defined as:

$$\begin{array}{c} \text{M1,}\;\mu_{\left[c\right]}=\beta_{1} \end{array}$$ (16)

and various, increasingly complex, multivariate models are fit, up to the most complex:

$$\begin{array}{ccc} \text{M25,}\;\mu_{\left[c\right]} & = & \beta_{1}+\beta_{2}\text{log}\left(N_{\left[c\right]}\right)+\beta_{3}\text{log}\left(P_{\left[c\right]}\right)+\beta_{4}\text{log}\left(I_{\left[c\right]}\right)+\beta_{5}\text{log}\left(G_{\left[c\right]}\right)+\beta_{6}\text{log}\left(H_{\left[c\right]}\right)\\ & & +\beta_{7}\text{log}\left(A_{\left[c\right]}^{W}\right)+\beta_{8}\text{log}\left(A_{\left[c\right]}^{B}\right)+\beta_{9}\text{log}\left(W_{\left[c\right]}^{W}\right)+\beta_{10}\text{log}\left(W_{\left[c\right]}^{B}\right) \end{array}$$ (17)

where: *N*_[*c*]_ is the total population in county *c*, *P*_[*c*]_ is the percentage of black individuals in county *c*, *I*_[*c*]_ is the median income of county *c*, *G*_[*c*]_ is the Gini coefficient of county *c*, *H*_[*c*]_ is the Google search data proxy for racist norms in county *c*, $A_{\left[c\right]}^{W}$ and $A_{\left[c\right]}^{B}$ are the assault-related arrest rates for whites and blacks, respectively, in county *c*, and $W_{\left[c\right]}^{W}$ and $W_{\left[c\right]}^{B}$ are the weapons-related arrest rates for whites and blacks, respectively, in county *c*. The covariates included in models M2–M24 are indicated in Tables 1 and 2 in the Results section.

Each element of the regression parameter vector is given a vague prior:

$$\begin{array}{c} \beta ∼\text{Cauchy}(0,5) \end{array}$$ (18)

and the scale parameter is given a weakly regularizing prior:

$$\begin{array}{c} \sigma ∼\text{Exponetial}(1) \end{array}$$ (19)

## Supporting Information

S1 File This file (Zip format) contains the raw data used in modeling, model code, and supplementary results tables with MCMC convergence diagnostics. (ZIP)

S2 File This file (Zip format) contains the LaTeXsource code for this paper. (ZIP)
