# Supplementary material for: A Multi-Level Bayesian Analysis of Racial Bias in Police Shootings at the County-Level in the United States, 2011–2014
Source: PLoS One. 2015 Nov 5;10(11):e0141854. doi: 10.1371/journal.pone.0141854 (PMC4634878; doi:10.1371/journal.pone.0141854)
Supplement: S1 File — (ZIP) [file pone.0141854.s001.zip › SupplementaryMaterials/Appendix.pdf]

Appendix: A Multi-Level Bayesian Analysis of Racial Bias in Police Shootings at the County-Level in the United States, 2011-2014

Cody T. Ross[1,*]
1 Department of Anthropology, University of California, Davis. Davis, California, USA.
∗ Corresponding Author
E-mail: ctross@ucdavis.edu

An Alternate Model of Race-Specific Crime Rates

In the main text, race-specific crime rates are always entered as simultaneous predictors in the models (see Tables 1 and 2 in the main text). As such, the rates of assaults or weapons-related arrests in the black population are always examined as predictors holding constant the corresponding rates in the white population. This model parameterization allows us to examine the effects of race-specific crime rates on racial bias in police shootings. However, there are questions that this model parameterization precludes. Most importantly, having an aggregated measure of crime rate allows one to test the questions: 1) does racial bias in police shooting increase in areas where crime is generally more prevalent? And, 2) as the difference of black crime rate minus white crime rate increases, does racial bias in police shootings increase?

As a robustness check, I present the results from two alternative model parameterizations in predicting the relative risk of being {unarmed, black, and shot by police} to being {unarmed, white, and shot by police}, based on including the sum and difference of race-specific crime rates in the model.

Methods

In each model in the main text where assault or weapons rate appears, the terms $\log(A^W_{[c]})$ and $\log(A^B_{[c]})$—the log assault-related arrest rates for whites and blacks, respectively—and, the terms $\log(W^W_{[c]})$ and $\log(W^B_{[c]})$—the log weapons-related arrest rates for whites and blacks, respectively—are replaced with the terms $\log(A_{[c]})$ and $A^*_{[c]}$ and/or $\log(W_{[c]})$ and $W^*_{[c]}$, respectively. In this case, $\log(A_{[c]})$ and $\log(W_{[c]})$ are the log aggregated crime rates, and $A^*_{[c]}$ and $W^*_{[c]}$, are the difference in race-specific crime rates, black minus white. Two ways of generating the aggregates are considered. In the first, the race-specific crime rates are directly summed, $A^W_{[c]} + A^B_{[c]}$. In the second, the race-specific counts of crimes are summed and divided by the sum of the race-specific population sizes.

Thus, we can—for example—rewrite $M25$ from the main text:

$$\begin{aligned}\text{M25, } \mu_{[c]} =& \beta_1 + \beta_2\log(N_{[c]}) + \beta_3\log(P_{[c]}) + \beta_4\log(I_{[c]}) + \beta_5\log(G_{[c]}) + \beta_6\log(H_{[c]}) \\ &+ \beta_7\log(A^W_{[c]}) + \beta_8\log(A^B_{[c]}) + \beta_9\log(W^W_{[c]}) + \beta_{10}\log(W^B_{[c]})\end{aligned}$$

as:

$$\begin{aligned}\text{M25}_1\text{, } \mu_{[c]} =& \beta_1 + \beta_2\log(N_{[c]}) + \beta_3\log(P_{[c]}) + \beta_4\log(I_{[c]}) + \beta_5\log(G_{[c]}) + \beta_6\log(H_{[c]}) \\ &+ \beta_7\log(A_{1[c]}) + \beta_8 A^*_{[c]} + \beta_9\log(W_{1[c]}) + \beta_{10} W^*_{[c]}\end{aligned}$$

and,

$$\begin{aligned}\text{M25}_2\text{, } \mu_{[c]} =& \beta_1 + \beta_2\log(N_{[c]}) + \beta_3\log(P_{[c]}) + \beta_4\log(I_{[c]}) + \beta_5\log(G_{[c]}) + \beta_6\log(H_{[c]}) \\ &+ \beta_7\log(A_{2[c]}) + \beta_8 A^*_{[c]} + \beta_9\log(W_{2[c]}) + \beta_{10} W^*_{[c]}\end{aligned}$$

where: $A_{[c]}$ and $W_{[c]}$ are the aggregated crime rates, with a subscript of 1 indicating that they were generated by directly summing the race-specific rates, and a subscript of 2 indicating that they were

generated by summing counts and dividing by summed population size. The other covariates included in models M1-M25 are unchanged from the main text. In these revised model, we also add models M26 and M27, where:

$$\text{M26, } \mu_{[c]} = \beta_1 + \beta_2 \log(N_{[c]}) + \beta_3 \log(A_{[c]})$$

and:

$$\text{M27, } \mu_{[c]} = \beta_1 + \beta_2 \log(N_{[c]}) + \beta_3 \log(W_{[c]})$$

Results and Discussion

Tables 1 and 2 present the results of the sensitivity analysis. The results of the main study hold qualitatively in this supplementary analysis. Parameterizing the model using an aggregated crime rate measure and a measure of difference in race-specific crime rates does not change the main interpretation of the data—that racial bias in police shootings is: 1) not associated with crime rates, and 2) not associated with race-specific crime rates.

[Table 1 about here.]

[Table 2 about here.]

List of Tables

1 Predictors of an increased county-level risk of being {black, unarmed, and shot by police} relative to being {white, unarmed, and shot by police}. Values are: posterior mean (posterior standard deviation) of the regression coefficients. The symbol *log* referes to the natural logarithm. *Pop* refers to absolute population size. *Pct. B.* refers to the percentage of the county population that is black. *Md. In.* refers to median income. *Gini* refers to the Gini index of inequality. *GRP* refers to the Google search racism proxy. *S. Ast.* and *D. Ast.* refer to the sum and difference in arrest rate for assualt, respectivley. *S. Wps.* and *S. Wps.* refer to the sum and difference in arrest rate for weapons violations. Posterior probabilty that a postive regression coeffcient is less than zero (or a negative one greater than zero) is color coded: yellow indicates a probability between 0.10 and 0.05, orange indicates a probability between 0.05 and 0.01, and red indicates a probability of 0.01 or less. *S. Ast.* and *S. Wps.* are calculated by summing the race-specific crime rates. 4

2 Predictors of an increased county-level risk of being {black, unarmed, and shot by police} relative to being {white, unarmed, and shot by police}. Values are: posterior mean (posterior standard deviation) of the regression coefficients. The symbol *log* referes to the natural logarithm. *Pop* refers to absolute population size. *Pct. B.* refers to the percentage of the county population that is black. *Md. In.* refers to median income. *Gini* refers to the Gini index of inequality. *GRP* refers to the Google search racism proxy. *S. Ast.* and *D. Ast.* refer to the sum and difference in arrest rate for assualt, respectivley. *S. Wps.* and *S. Wps.* refer to the sum and difference in arrest rate for weapons violations. Posterior probabilty that a postive regression coeffcient is less than zero (or a negative one greater than zero) is color coded: yellow indicates a probability between 0.10 and 0.05, orange indicates a probability between 0.05 and 0.01, and red indicates a probability of 0.01 or less. *S. Ast.* and *S. Wps.* are calculated by summing the counts of race-specific crimes and dividing by the sum of the race-specific population sizes. 5

Table 1. Predictors of an increased county-level risk of being {black, unarmed, and shot by police} relative to being {white, unarmed, and shot by police}. Values are: posterior mean (posterior standard deviation) of the regression coefficients. The symbol *log* referes to the natural logarithm. *Pop* refers to absolute population size. *Pct. B.* refers to the percentage of the county population that is black. *Md. In.* refers to median income. *Gini* refers to the Gini index of inequality. *GRP* refers to the Google search racism proxy. *S. Ast.* and *D. Ast.* refer to the sum and difference in arrest rate for assualt, respectivley. *S. Wps.* and *S. Wps.* refer to the sum and difference in arrest rate for weapons violations. Posterior probabilty that a postive regression coeffcient is less than zero (or a negative one greater than zero) is color coded: yellow indicates a probability between 0.10 and 0.05, orange indicates a probability between 0.05 and 0.01, and red indicates a probability of 0.01 or less. *S. Ast.* and *S. Wps.* are calculated by summing the race-specific crime rates.

Model	Intercept	SD	log(Pop)	log(Pct. B.)	log(Gini)	log(Md. In.)	log(GRP)	log(S. Ast.)	(D. Ast.)	log(S. Wps.)	(D. Wps.)
M7	1.42 (0.11)	0.02 (0.01)	0.1 (0.05)					-0.02 (0.07)	-0.01 (0.06)		
M8	1.4 (0.1)	0.09 (0.04)	0.08 (0.05)							0.06 (0.08)	-0.02 (0.05)
M12	1.55 (0.19)	0.03 (0.02)	0.08 (0.05)	0.06 (0.06)				-0.03 (0.09)	-0.01 (0.06)		
M13	1.51 (0.16)	0.11 (0.03)	0.07 (0.05)	0.06 (0.06)						0.04 (0.08)	-0.02 (0.05)
M16	2.08 (0.4)	0.1 (0.03)	0.12 (0.05)	0.04 (0.05)		-0.39 (0.26)		-0.05 (0.07)	-0.02 (0.05)		
M17	2.02 (0.45)	0.08 (0.03)	0.09 (0.05)	0.05 (0.05)		-0.37 (0.3)				-0.01 (0.08)	-0.01 (0.04)
M19	2.2 (0.61)	0.1 (0.02)	0.07 (0.06)	0.03 (0.06)	0.99 (0.82)			-0.04 (0.07)	0.02 (0.06)		
M20	2.28 (0.61)	0.07 (0.02)	0.06 (0.05)	0.05 (0.06)	0.95 (0.8)					-0.01 (0.09)	-0.01 (0.05)
M21	1.51 (1.41)	0.09 (0.03)	0.11 (0.06)	0.05 (0.07)		-0.35 (0.3)	0.12 (0.3)	-0.03 (0.08)	-0.01 (0.06)		
M22	1.5 (1.27)	0.1 (0.03)	0.11 (0.06)	0.04 (0.06)		-0.39 (0.31)	0.13 (0.28)			-0.02 (0.09)	-0.01 (0.05)
M23	1.68 (1.29)	0.06 (0.04)	0.06 (0.05)	0.03 (0.05)	0.38 (0.74)		0.03 (0.24)	0.01 (0.08)	-0.03 (0.04)		
M24	1.98 (1.48)	0.06 (0.03)	0.06 (0.05)	0.05 (0.06)	0.99 (0.81)		0.08 (0.3)			0.02 (0.09)	-0.02 (0.05)
M25	2 (1.46)	0.08 (0.02)	0.1 (0.07)	0.03 (0.06)	0.68 (0.97)	-0.29 (0.34)	0.11 (0.28)	0.01 (0.08)	-0.02 (0.05)	-0.02 (0.08)	-0.01 (0.07)
M26	1.42 (0.09)	0.1 (0.04)	0.11 (0.05)							-0.03 (0.06)	
M27	1.39 (0.09)	0.1 (0.04)	0.09 (0.05)					0.03 (0.08)			

Table 2. Predictors of an increased county-level risk of being {black, unarmed, and shot by police} relative to being {white, unarmed, and shot by police}. Values are: posterior mean (posterior standard deviation) of the regression coefficients. The symbol *log* referes to the natural logarithm. *Pop* refers to absolute population size. *Pct. B.* refers to the percentage of the county population that is black. *Md. In.* refers to median income. *Gini* refers to the Gini index of inequality. *GRP* refers to the Google search racism proxy. *S. Ast.* and *D. Ast.* refer to the sum and difference in arrest rate for assualt, respectivley. *S. Wps.* and *S. Wps.* refer to the sum and difference in arrest rate for weapons violations. Posterior probabilty that a postive regression coeffcient is less than zero (or a negative one greater than zero) is color coded: yellow indicates a probability between 0.10 and 0.05, orange indicates a probability between 0.05 and 0.01, and red indicates a probability of 0.01 or less. *S. Ast.* and *S. Wps.* are calculated by summing the counts of race-specific crimes and dividing by the sum of the race-specific population sizes.

Model	Intercept	SD	log(Pop)	log(Pct. B.)	log(Gini)	log(Md. In.)	log(GRP)	log(S. Ast.)	(D. Ast.)	log(S. Wps.)	(D. Wps.)
M7	1.41 (0.1)	0.06 (0.05)	0.09 (0.04)					-0.01 (0.08)	-0.01 (0.06)		
M8	1.37 (0.11)	0.04 (0.04)	0.1 (0.05)							0.01 (0.09)	0.01 (0.04)
M12	1.61 (0.16)	0.06 (0.02)	0.08 (0.05)	0.08 (0.06)				-0.03 (0.08)	-0.02 (0.05)		
M13	1.53 (0.16)	0.08 (0.03)	0.08 (0.04)	0.06 (0.06)						0.02 (0.09)	-0.01 (0.04)
M16	2.05 (0.44)	0.04 (0.04)	0.1 (0.05)	0.05 (0.06)		-0.36 (0.31)		-0.05 (0.08)	-0.02 (0.05)		
M17	2.05 (0.47)	0.09 (0.04)	0.11 (0.05)	0.05 (0.06)		-0.38 (0.31)				-0.01 (0.1)	-0.01 (0.04)
M19	2.43 (0.65)	0.06 (0.04)	0.05 (0.05)	0.06 (0.06)	1.11 (0.81)			-0.03 (0.08)	-0.02 (0.05)		
M20	2.13 (0.68)	0.08 (0.03)	0.06 (0.05)	0.05 (0.06)	0.77 (0.89)					0.02 (0.09)	-0.02 (0.04)
M21	1.47 (1.19)	0.08 (0.04)	0.11 (0.06)	0.05 (0.05)		-0.3 (0.29)	0.13 (0.26)	-0.03 (0.08)	-0.03 (0.06)		
M22	1.58 (1.58)	0.09 (0.03)	0.1 (0.06)	0.04 (0.07)		-0.33 (0.33)	0.09 (0.32)			0.03 (0.09)	-0.03 (0.04)
M23	1.77 (1.49)	0.06 (0.03)	0.06 (0.05)	0.04 (0.06)	0.96 (0.82)		0.12 (0.31)	0 (0.08)	-0.02 (0.05)		
M24	1.77 (1.3)	0.07 (0.03)	0.08 (0.05)	0.03 (0.06)	1.07 (0.79)		0.14 (0.27)			0.01 (0.09)	-0.02 (0.04)
M25	1.69 (1.5)	0.12 (0.04)	0.08 (0.07)	0.03 (0.06)	0.55 (1.09)	-0.2 (0.3)	0.13 (0.26)	0.02 (0.09)	-0.02 (0.05)	-0.04 (0.09)	-0.01 (0.06)
M26	1.41 (0.08)	0.07 (0.03)	0.11 (0.04)					-0.03 (0.07)			
M27	1.37 (0.07)	0.09 (0.04)	0.09 (0.04)							0.04 (0.08)	
