# Supplementary material for: A Multi-Level Bayesian Analysis of Racial Bias in Police Shootings at the County-Level in the United States, 2011–2014
Source: PLoS One. 2015 Nov 5;10(11):e0141854. doi: 10.1371/journal.pone.0141854 (PMC4634878; doi:10.1371/journal.pone.0141854)
Supplement: S2 File — (ZIP) [file pone.0141854.s002.zip › LaTeXBuild/Figures/RR-Cater-HAU-eps-converted-to.pdf]

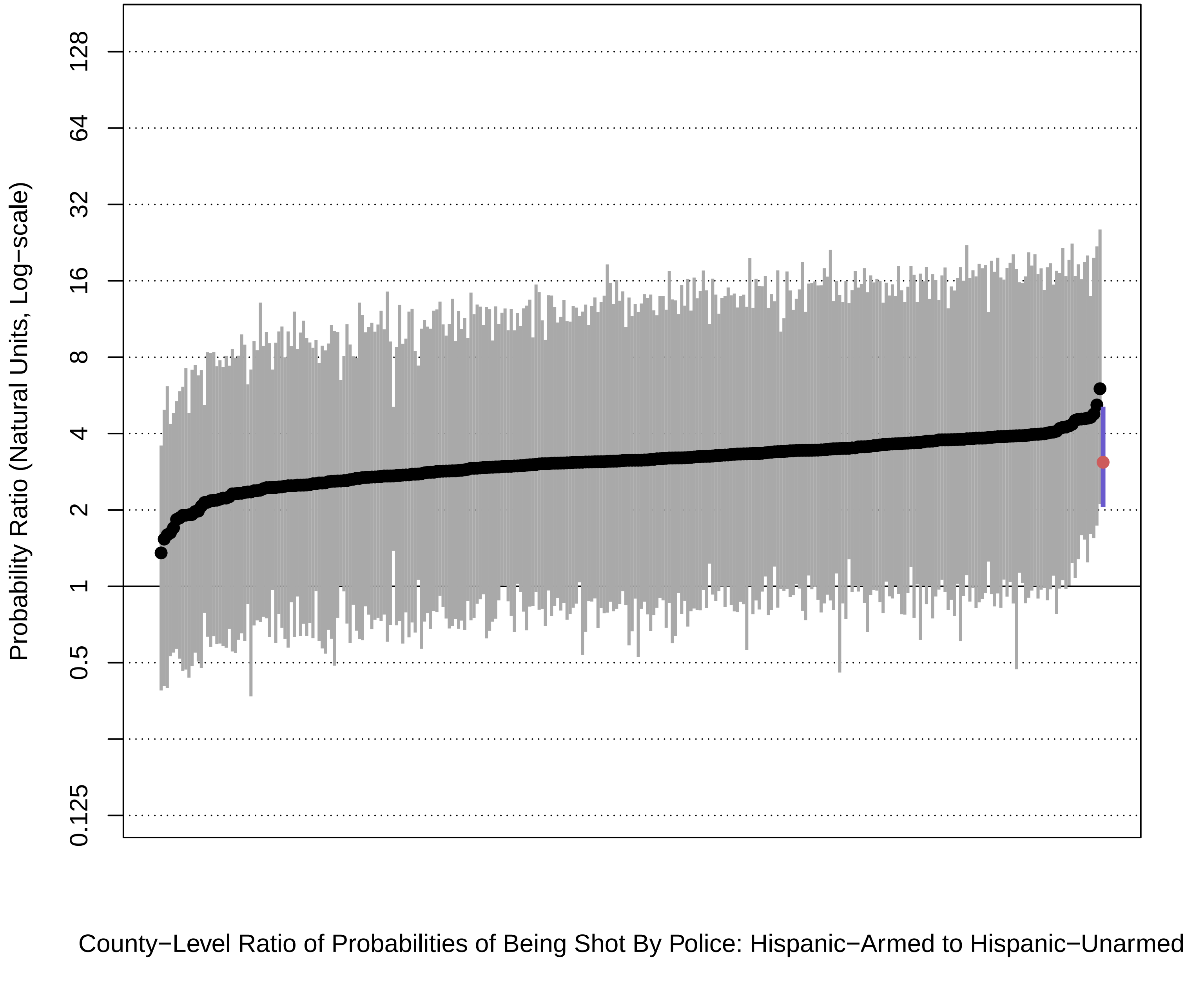
Probability Ratio (Natural Units, Log−scale)
128
64
32
16
8
4
2
1
0.5
0.125
County−Level Ratio of Probabilities of Being Shot By Police: Hispanic−Armed to Hispanic−Unarmed
