# Supplementary figures and images for: A Multi-Level Bayesian Analysis of Racial Bias in Police Shootings at the County-Level in the United States, 2011–2014
Source: PLoS One. 2015 Nov 5;10(11):e0141854. doi: 10.1371/journal.pone.0141854 (PMC4634878; doi:10.1371/journal.pone.0141854)

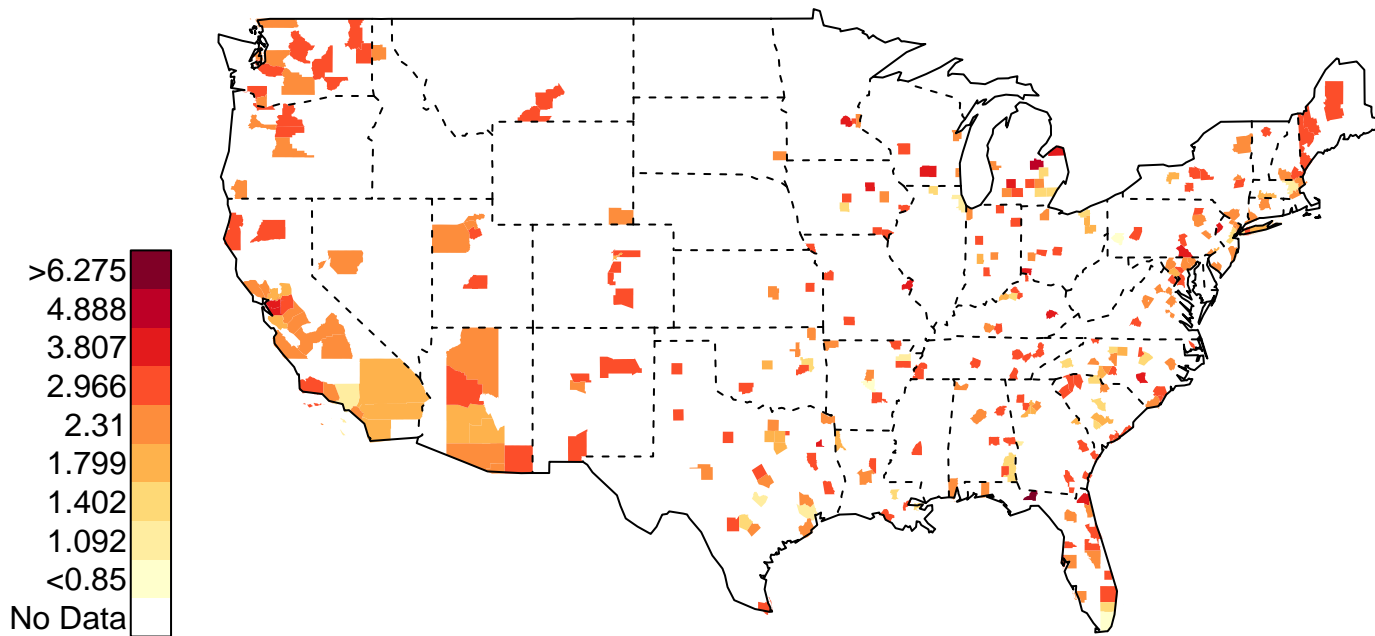

Supplement: S2 File — (ZIP) [file pone.0141854.s002.zip › LaTeXBuild/Figures/Map-BAU-eps-converted-to.pdf]

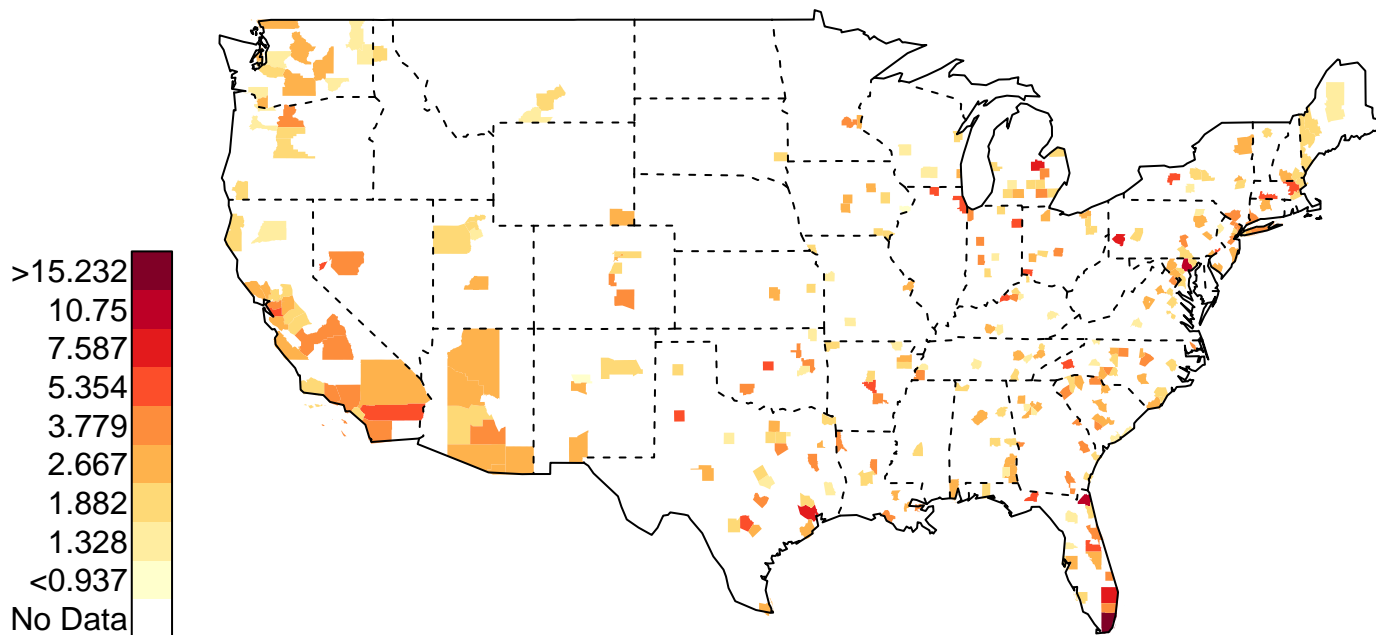

Supplement: S2 File — (ZIP) [file pone.0141854.s002.zip › LaTeXBuild/Figures/Map-BA-WA-eps-converted-to.pdf]

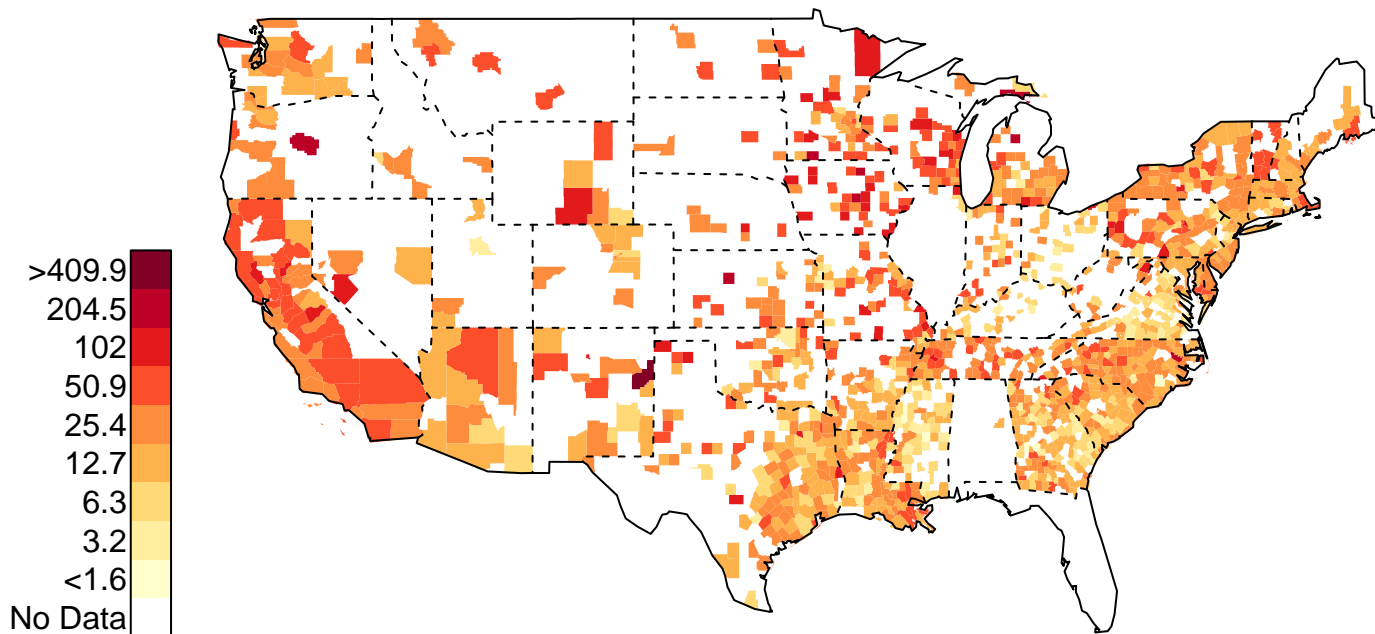

Supplement: S2 File — (ZIP) [file pone.0141854.s002.zip › LaTeXBuild/Figures/Map-BlackAssaultRate-eps-converted-to.pdf]

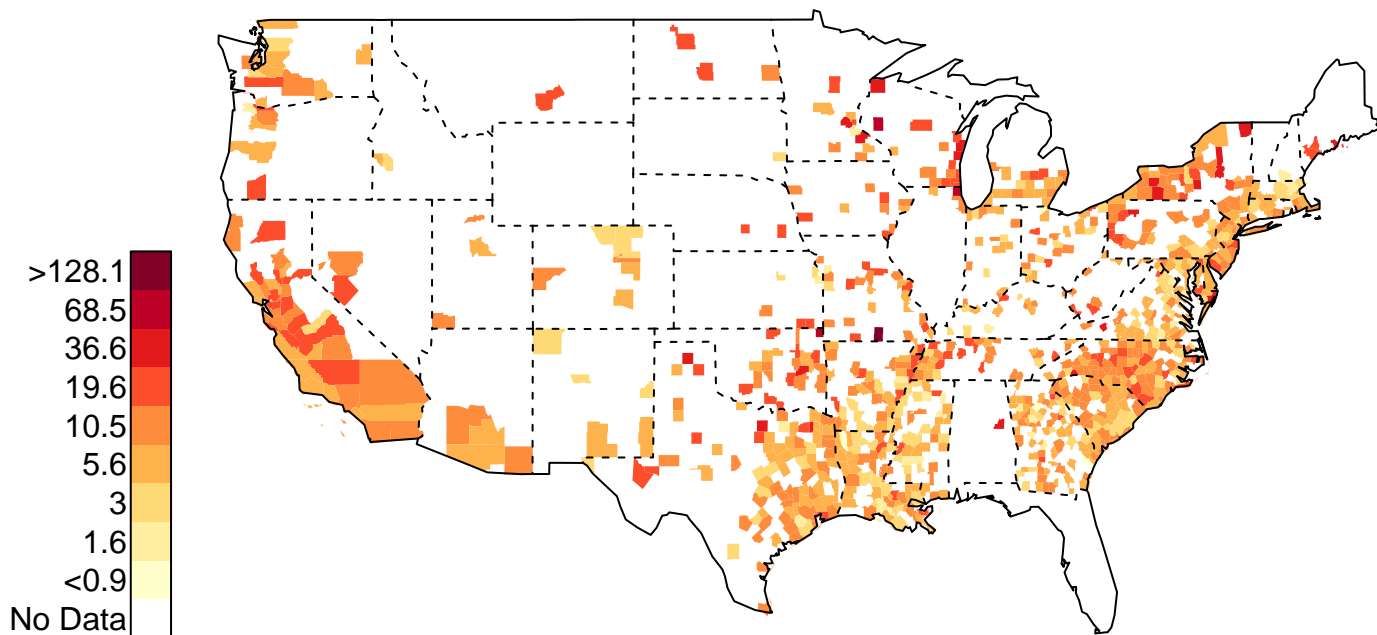

Supplement: S2 File — (ZIP) [file pone.0141854.s002.zip › LaTeXBuild/Figures/Map-BlackWeaponsRate-eps-converted-to.pdf]

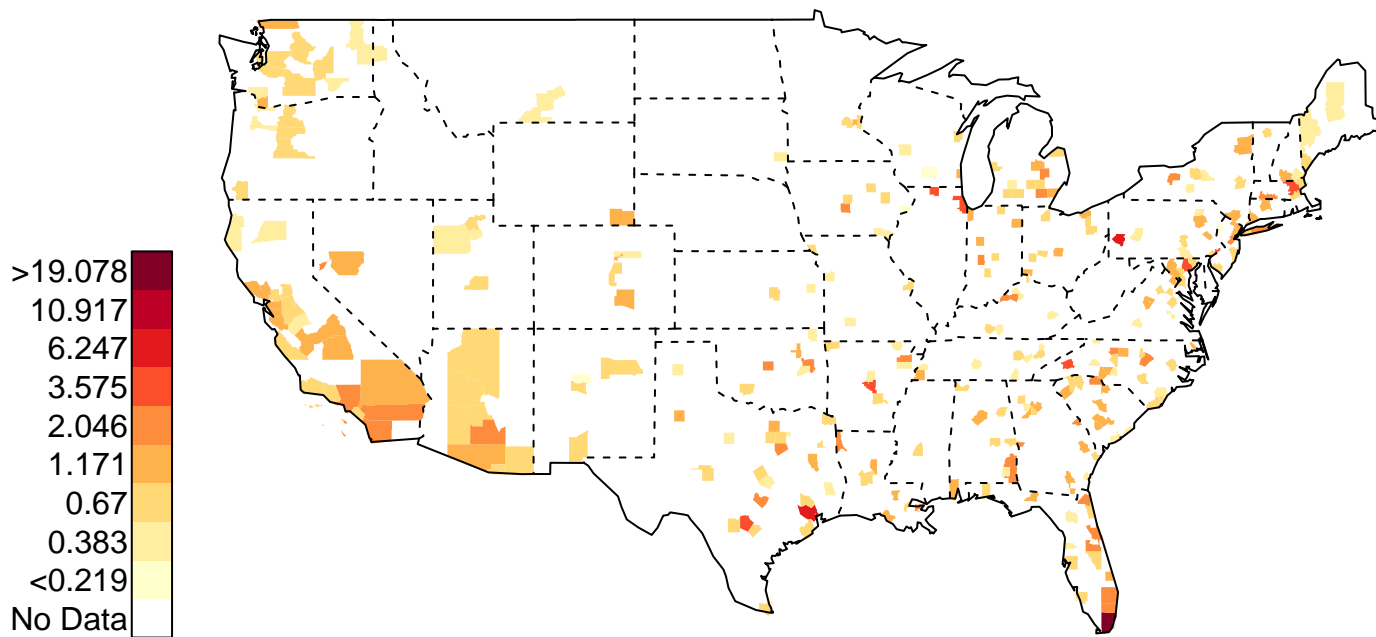

Supplement: S2 File — (ZIP) [file pone.0141854.s002.zip › LaTeXBuild/Figures/Map-BU-WA-eps-converted-to.pdf]

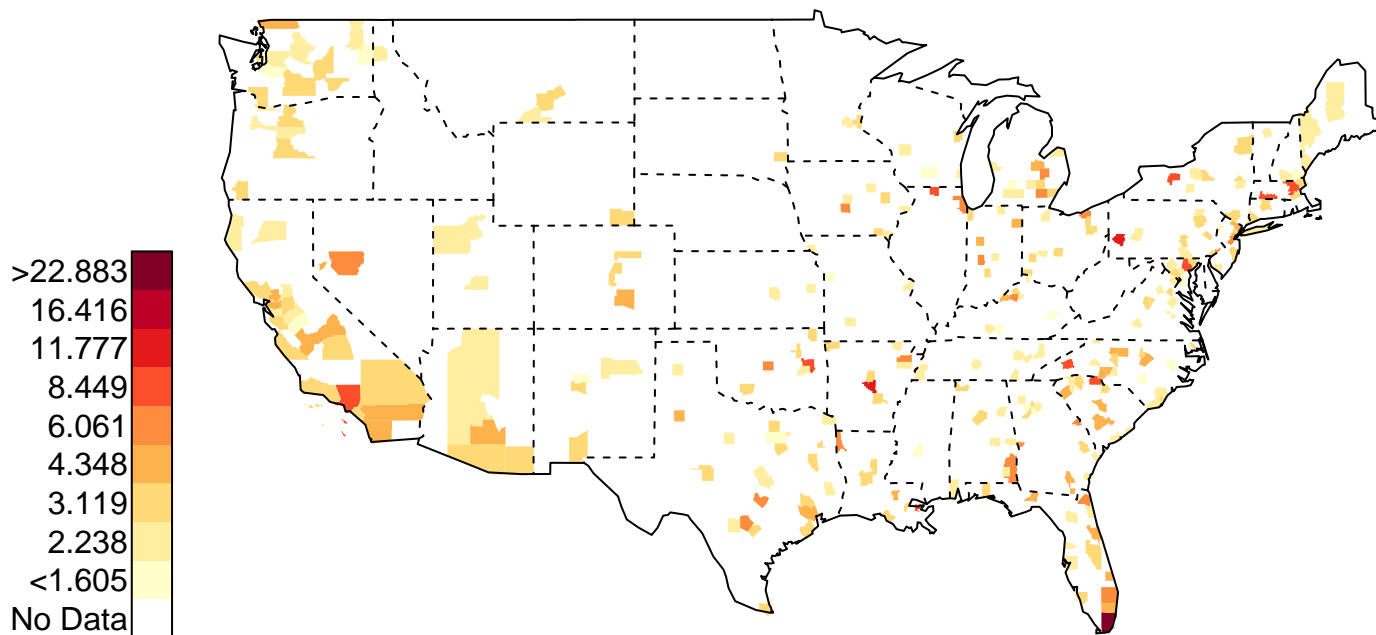

Supplement: S2 File — (ZIP) [file pone.0141854.s002.zip › LaTeXBuild/Figures/Map-BU-WU-eps-converted-to.pdf]

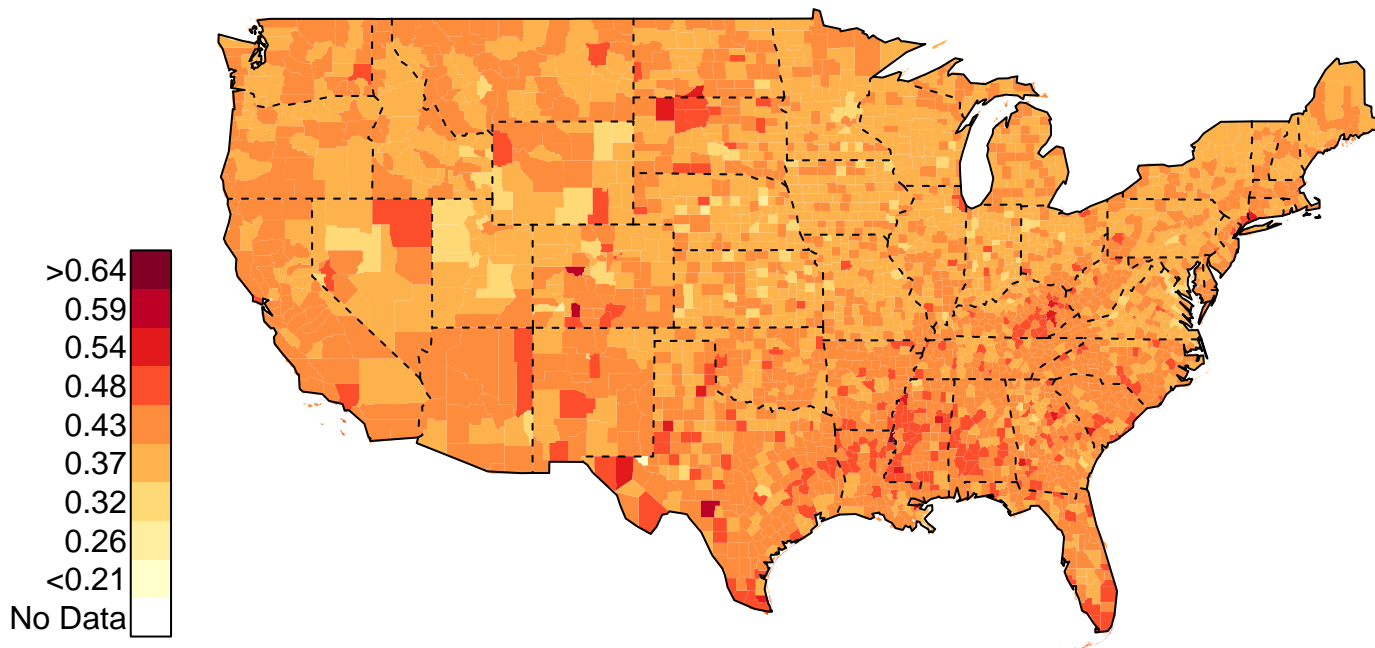

Supplement: S2 File — (ZIP) [file pone.0141854.s002.zip › LaTeXBuild/Figures/Map-Gini-eps-converted-to.pdf]

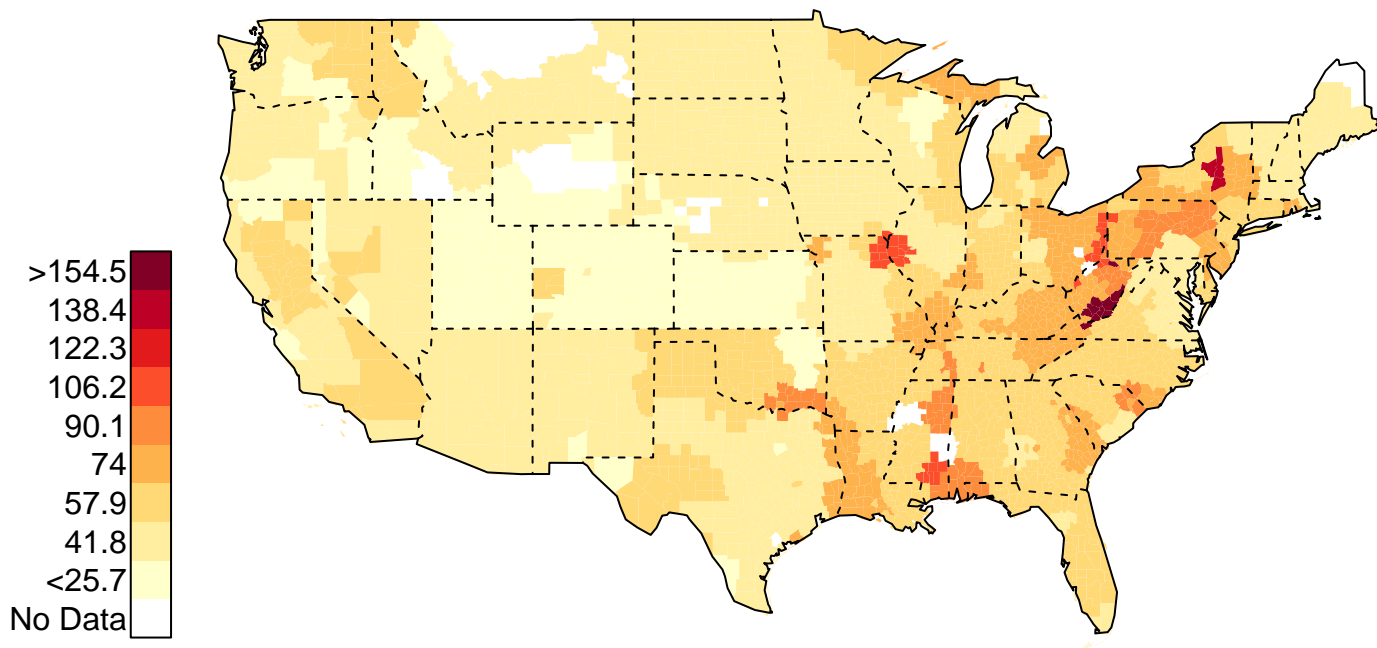

Supplement: S2 File — (ZIP) [file pone.0141854.s002.zip › LaTeXBuild/Figures/Map-Google-eps-converted-to.pdf]

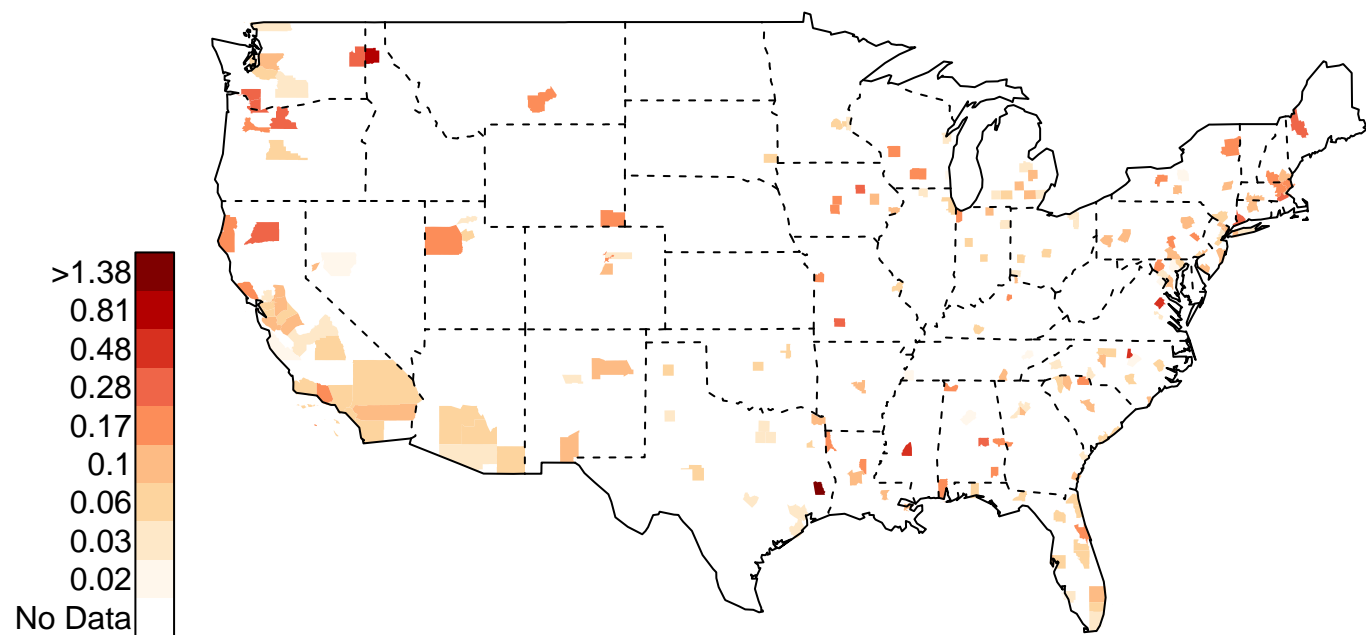

Supplement: S2 File — (ZIP) [file pone.0141854.s002.zip › LaTeXBuild/Figures/Map-Hate-PC-eps-converted-to.pdf]

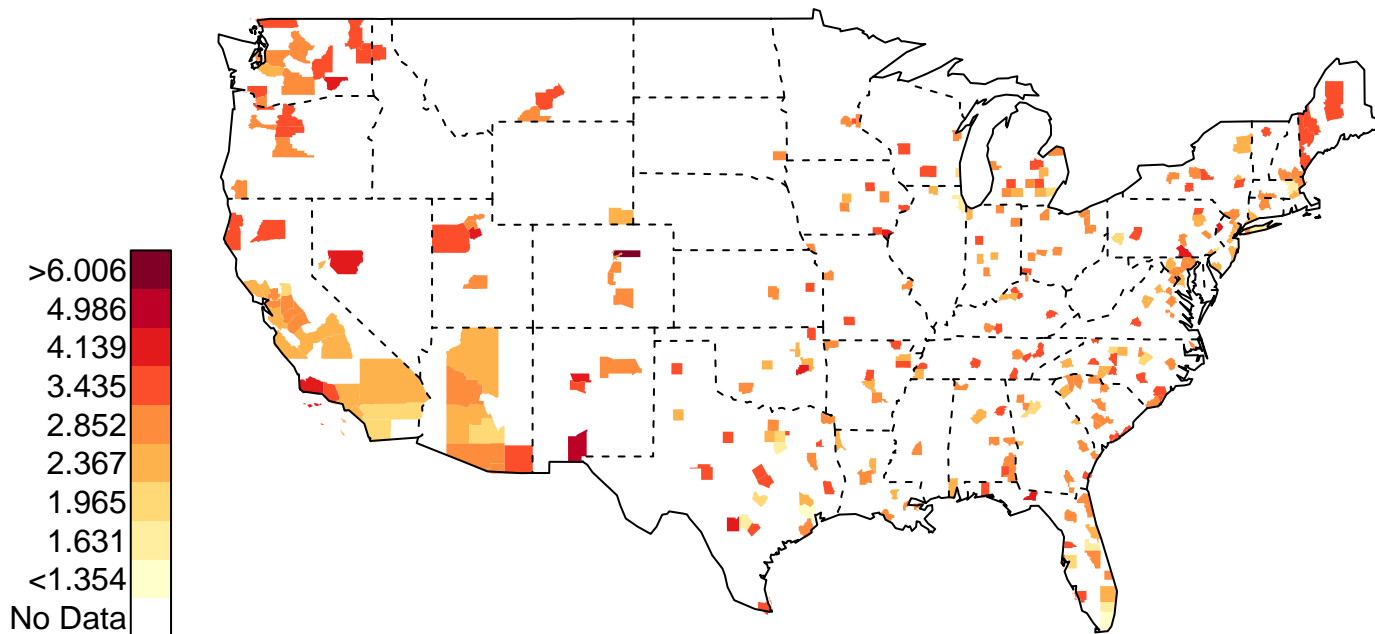

Supplement: S2 File — (ZIP) [file pone.0141854.s002.zip › LaTeXBuild/Figures/Map-HAU-eps-converted-to.pdf]

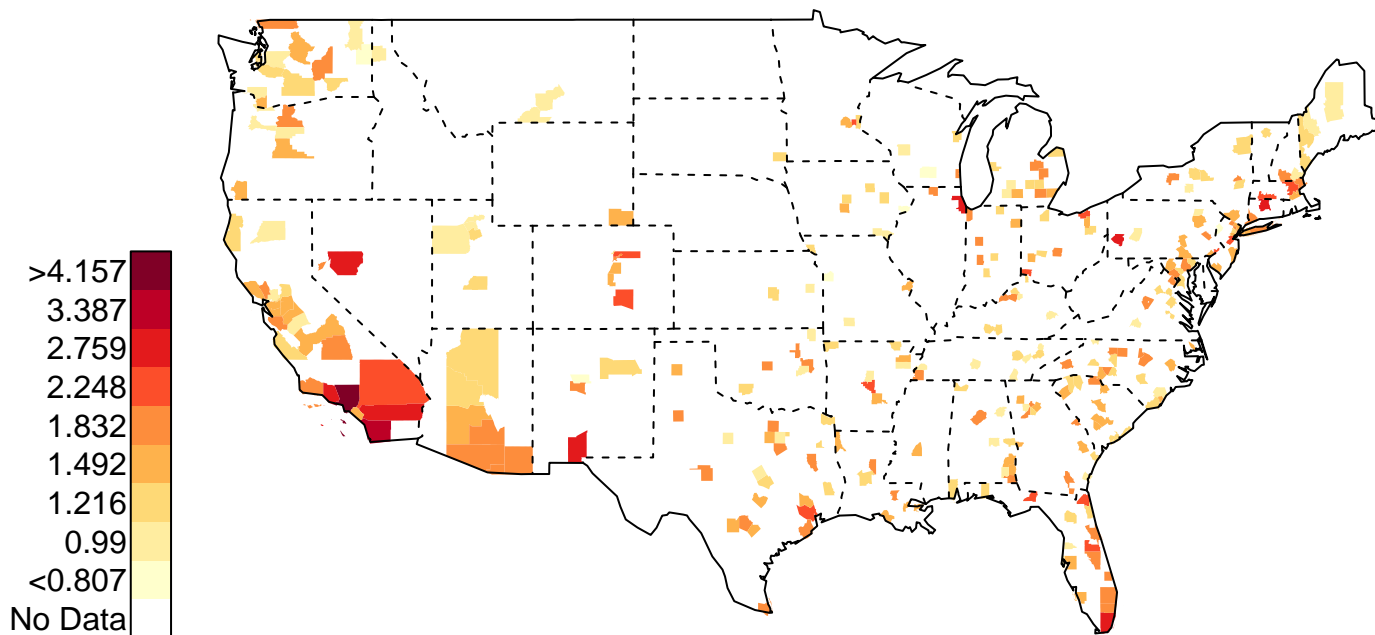

Supplement: S2 File — (ZIP) [file pone.0141854.s002.zip › LaTeXBuild/Figures/Map-HA-WA-eps-converted-to.pdf]

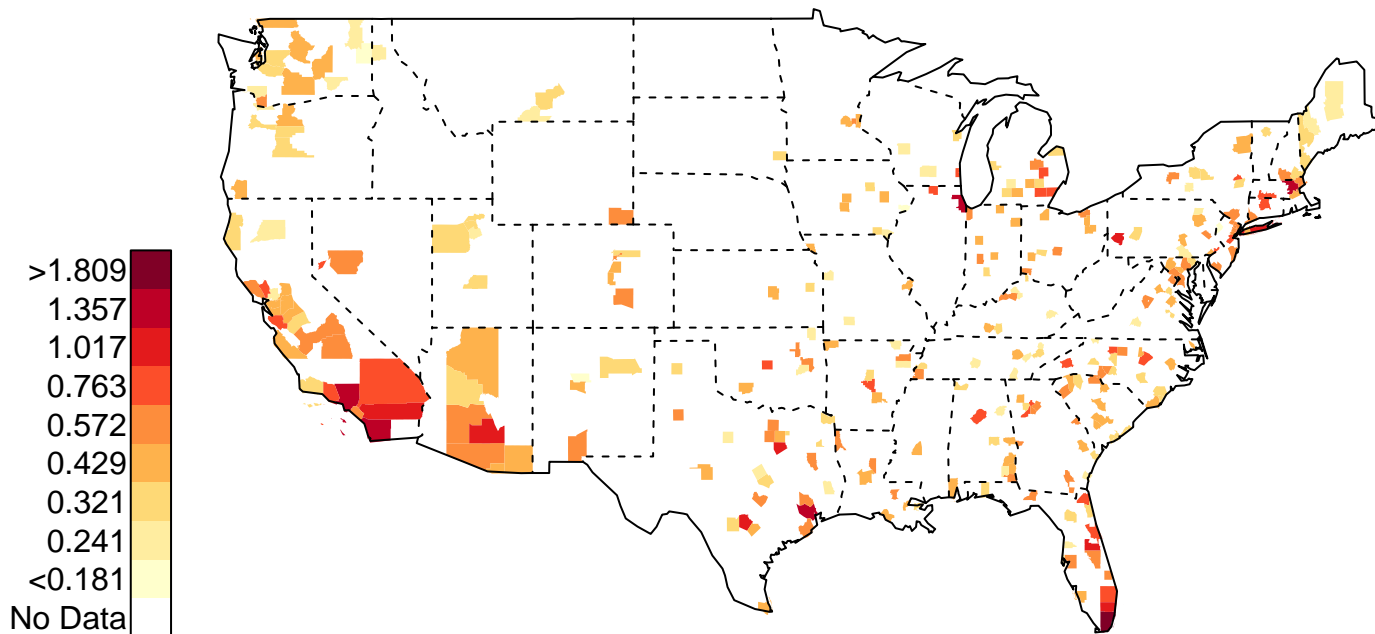

Supplement: S2 File — (ZIP) [file pone.0141854.s002.zip › LaTeXBuild/Figures/Map-HU-WA-eps-converted-to.pdf]

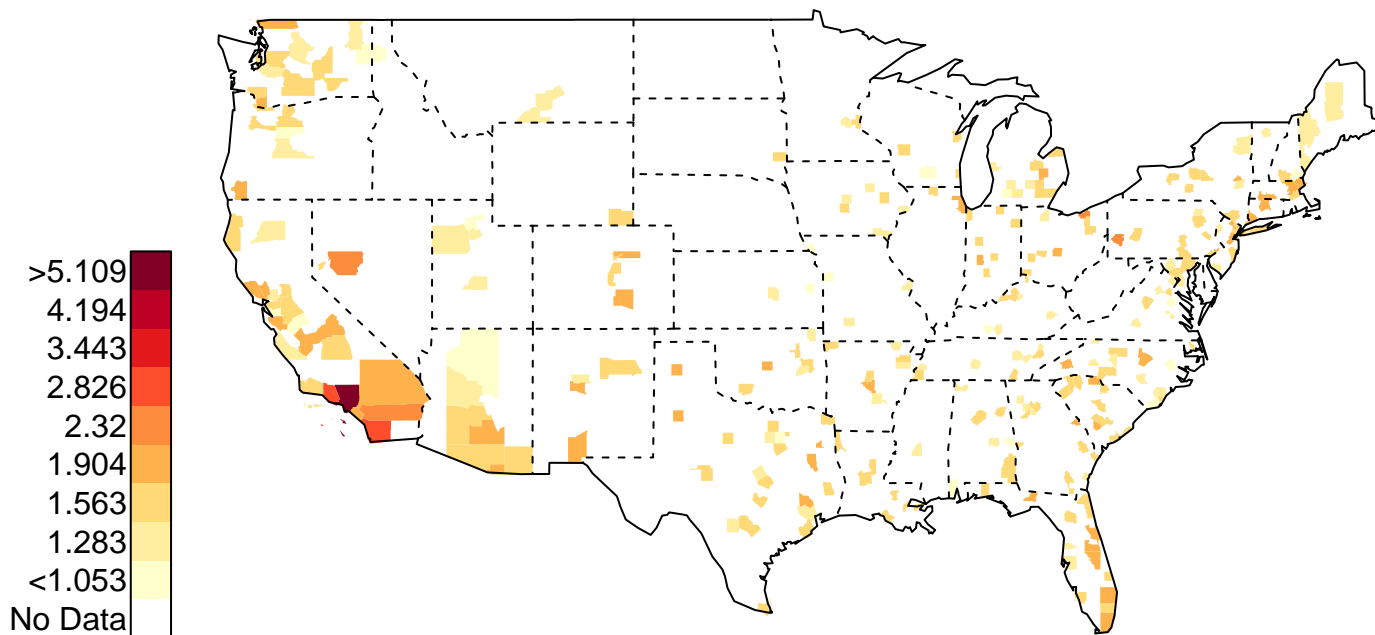

Supplement: S2 File — (ZIP) [file pone.0141854.s002.zip › LaTeXBuild/Figures/Map-HU-WU-eps-converted-to.pdf]

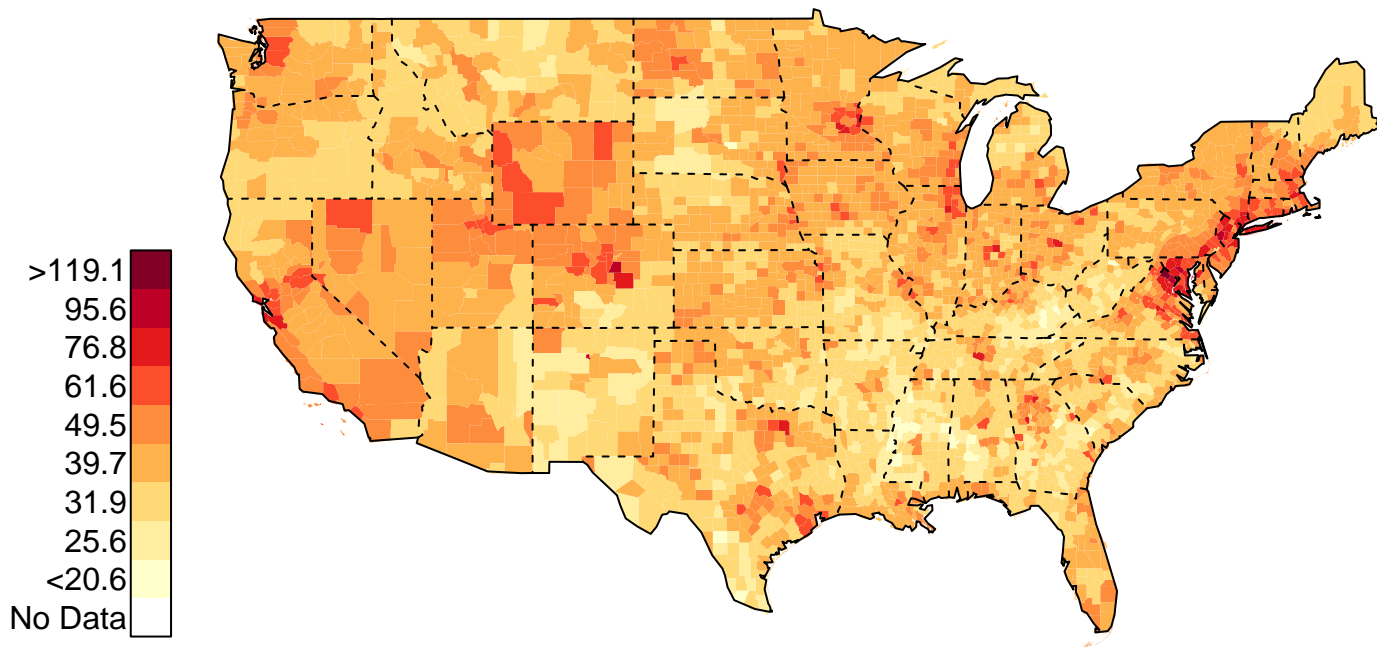

Supplement: S2 File — (ZIP) [file pone.0141854.s002.zip › LaTeXBuild/Figures/Map-MedianIncome10000-eps-converted-to.pdf]

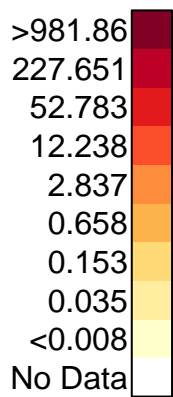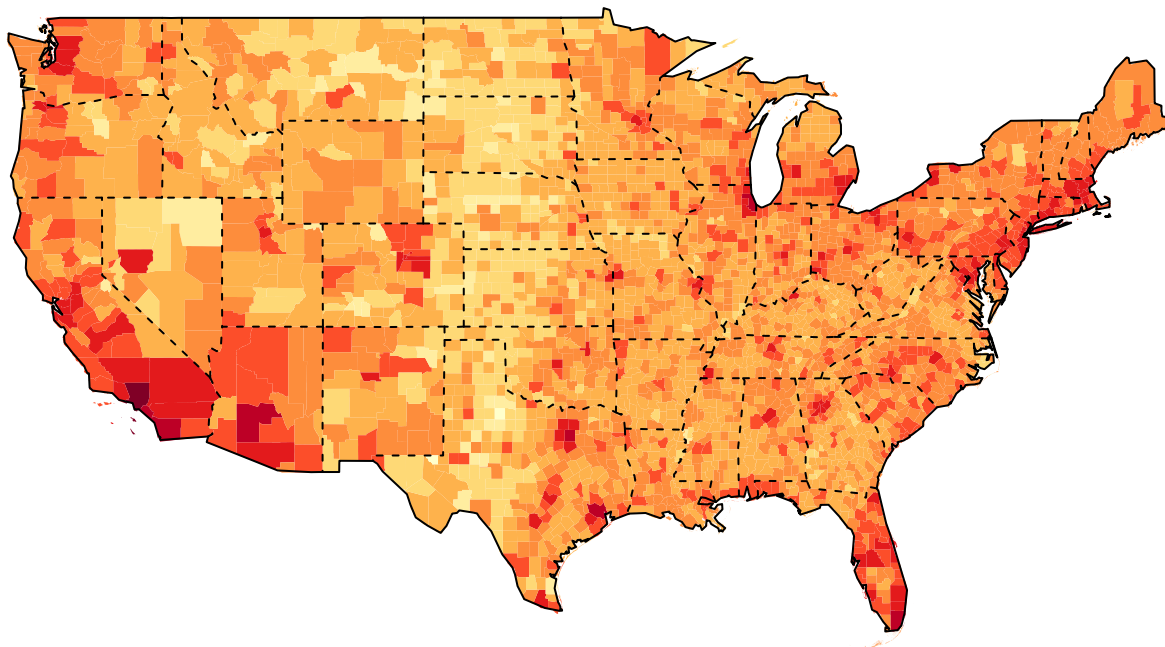

Supplement: S2 File — (ZIP) [file pone.0141854.s002.zip › LaTeXBuild/Figures/Map-Pop10000-eps-converted-to.pdf]

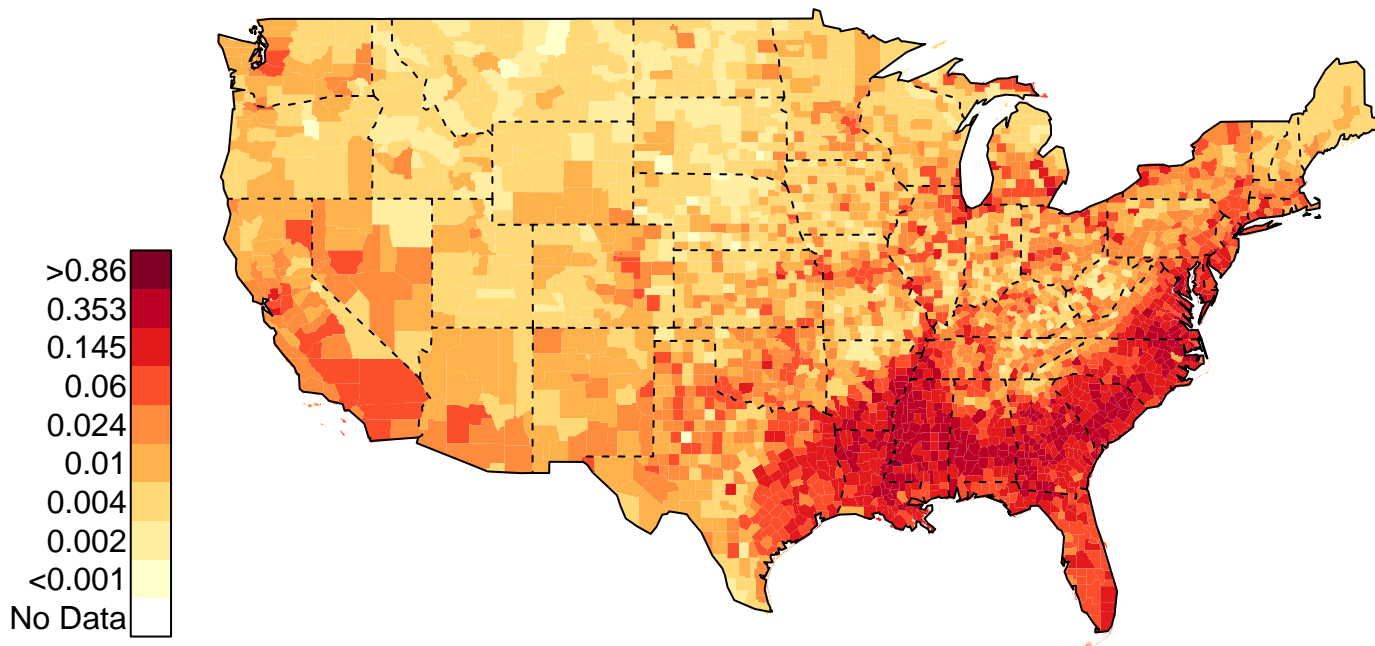

Supplement: S2 File — (ZIP) [file pone.0141854.s002.zip › LaTeXBuild/Figures/Map-PopRatio-eps-converted-to.pdf]

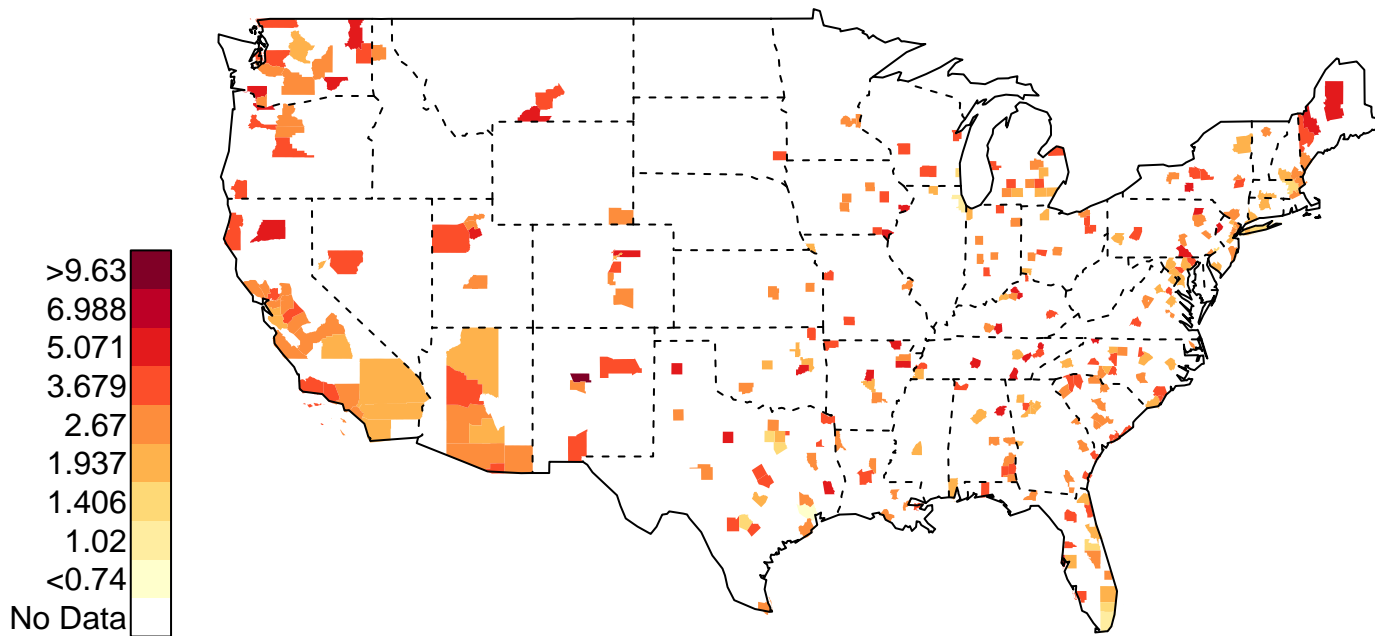

Supplement: S2 File — (ZIP) [file pone.0141854.s002.zip › LaTeXBuild/Figures/Map-WAU-eps-converted-to.pdf]

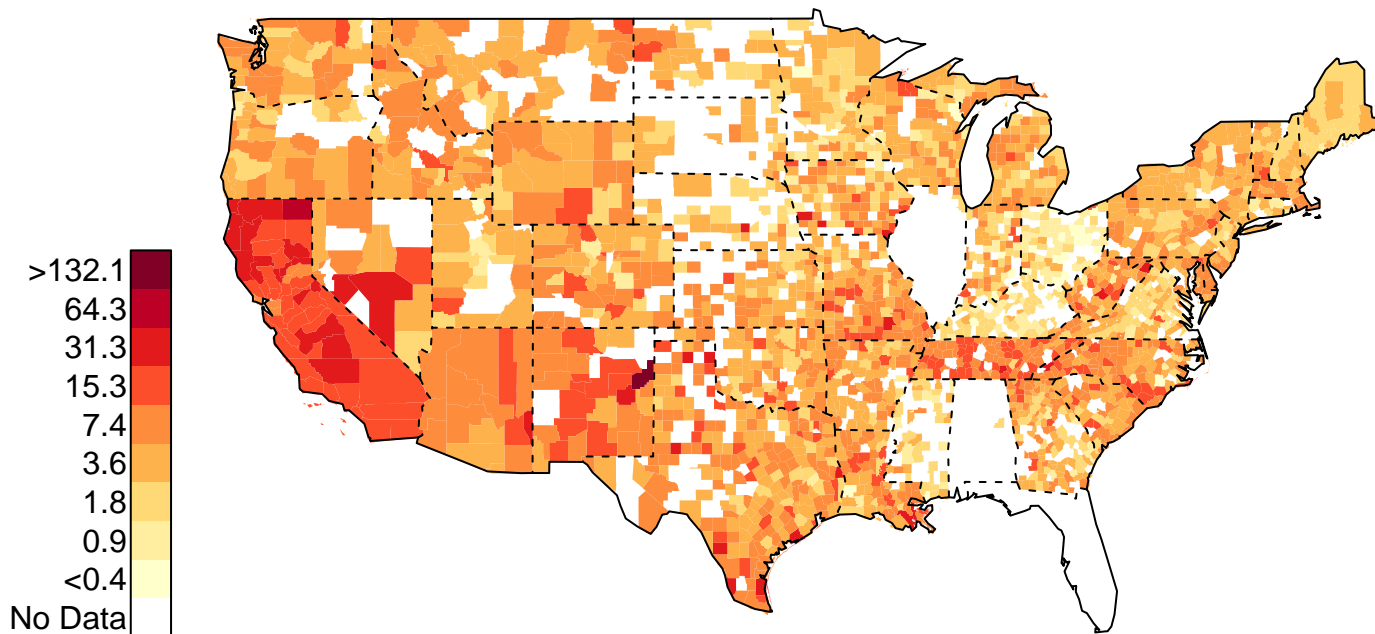

Supplement: S2 File — (ZIP) [file pone.0141854.s002.zip › LaTeXBuild/Figures/Map-WhiteAssaultRate-eps-converted-to.pdf]

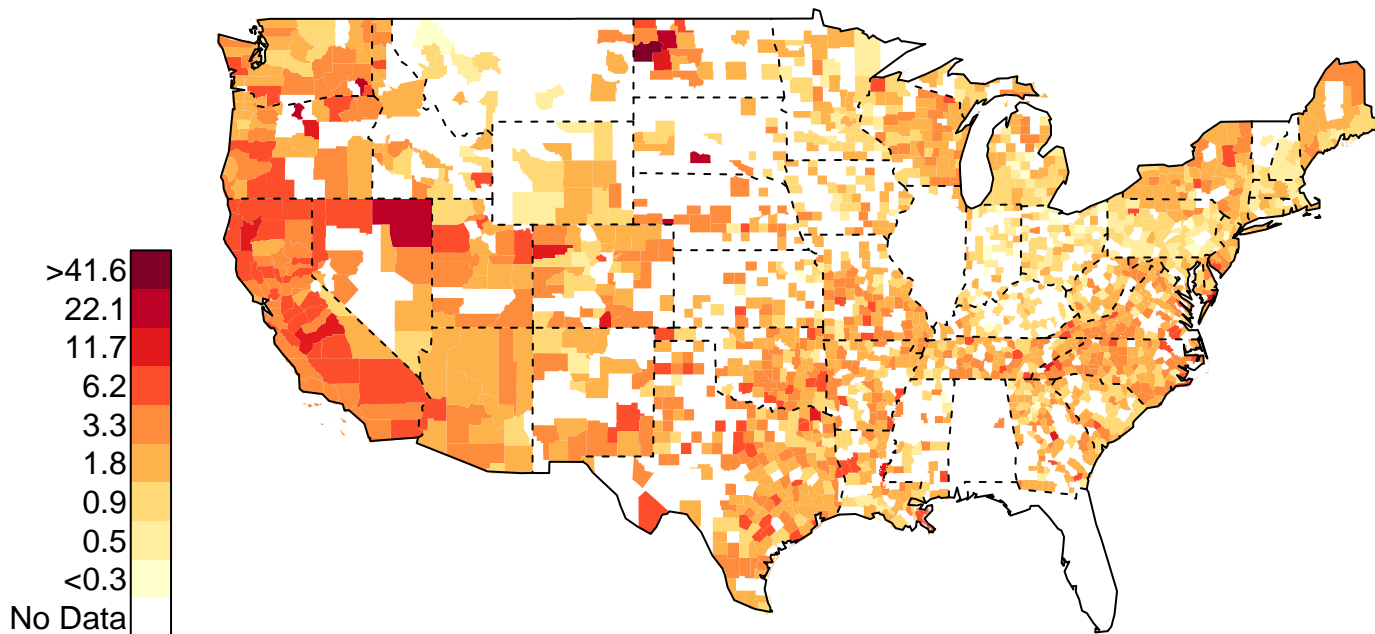

Supplement: S2 File — (ZIP) [file pone.0141854.s002.zip › LaTeXBuild/Figures/Map-WhiteWeaponsRate-eps-converted-to.pdf]

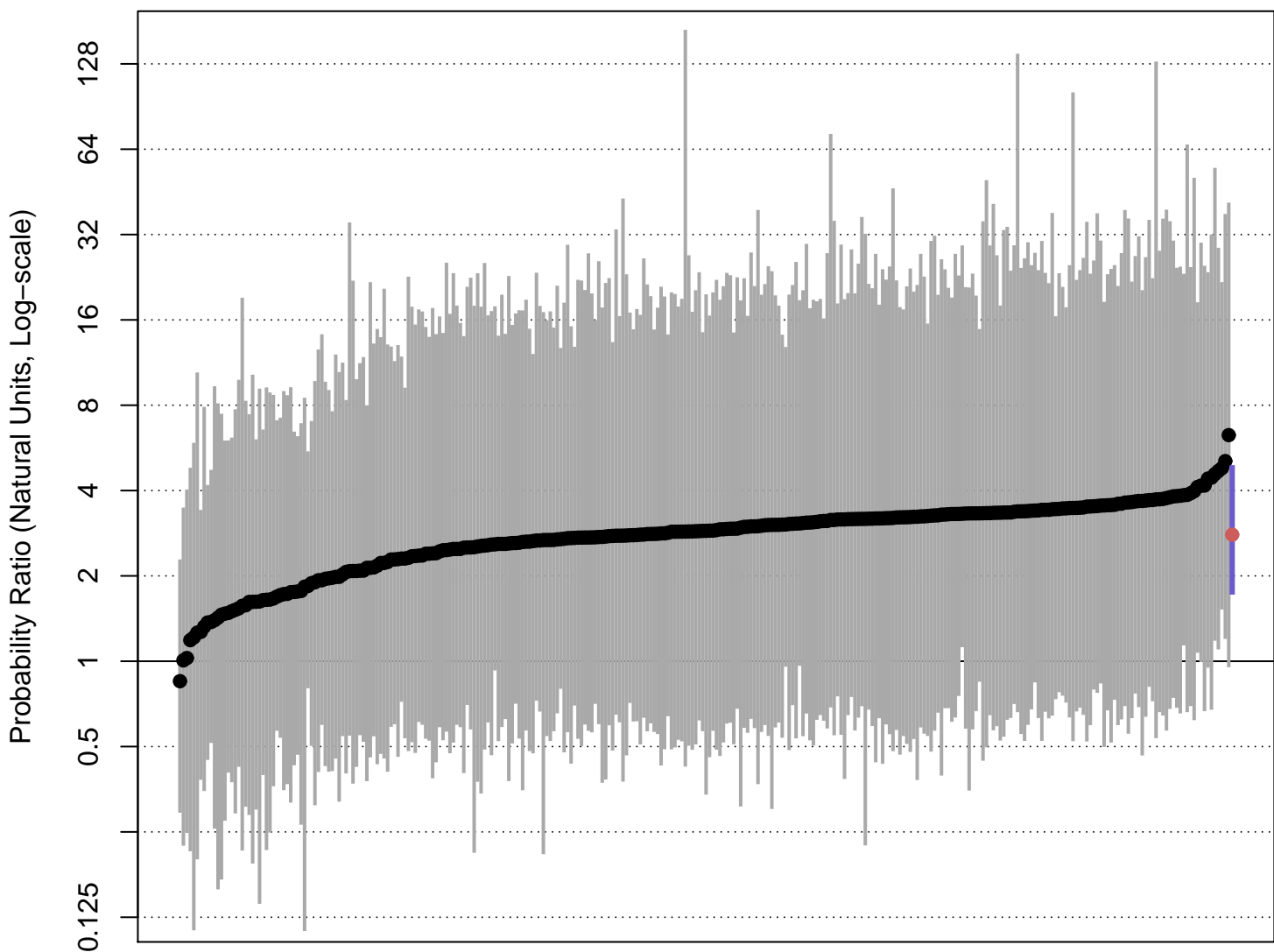

County-Level Ratio of Probabilities of Being Shot By Police: Black-Armed to Black-Unarmed

Supplement: S2 File — (ZIP) [file pone.0141854.s002.zip › LaTeXBuild/Figures/RR-Cater-BAU-eps-converted-to.pdf]

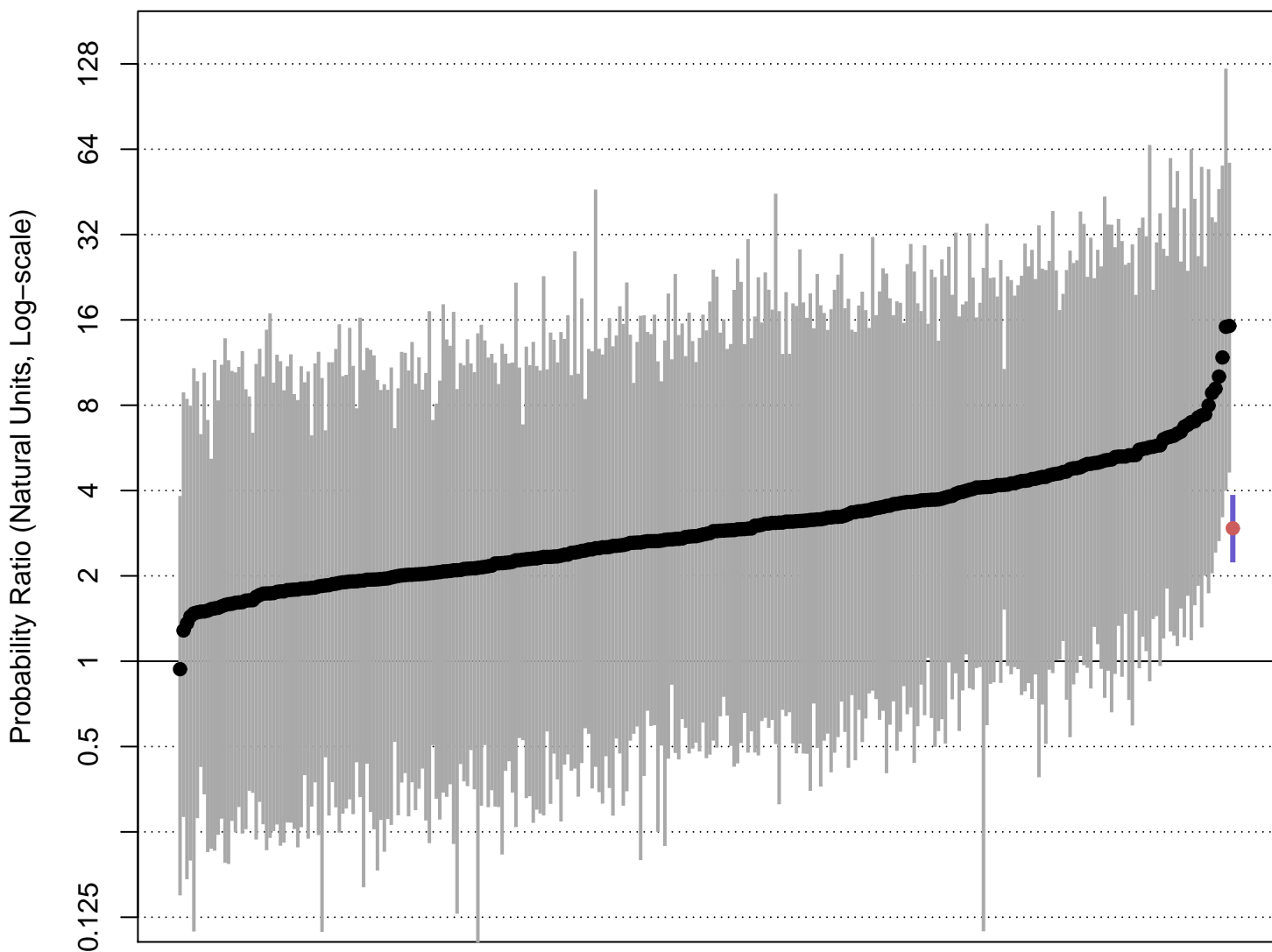

County-Level Ratio of Probabilities of Being Shot By Police: Black-Armed to White-Armed

Supplement: S2 File — (ZIP) [file pone.0141854.s002.zip › LaTeXBuild/Figures/RR-Cater-BA-WA-eps-converted-to.pdf]

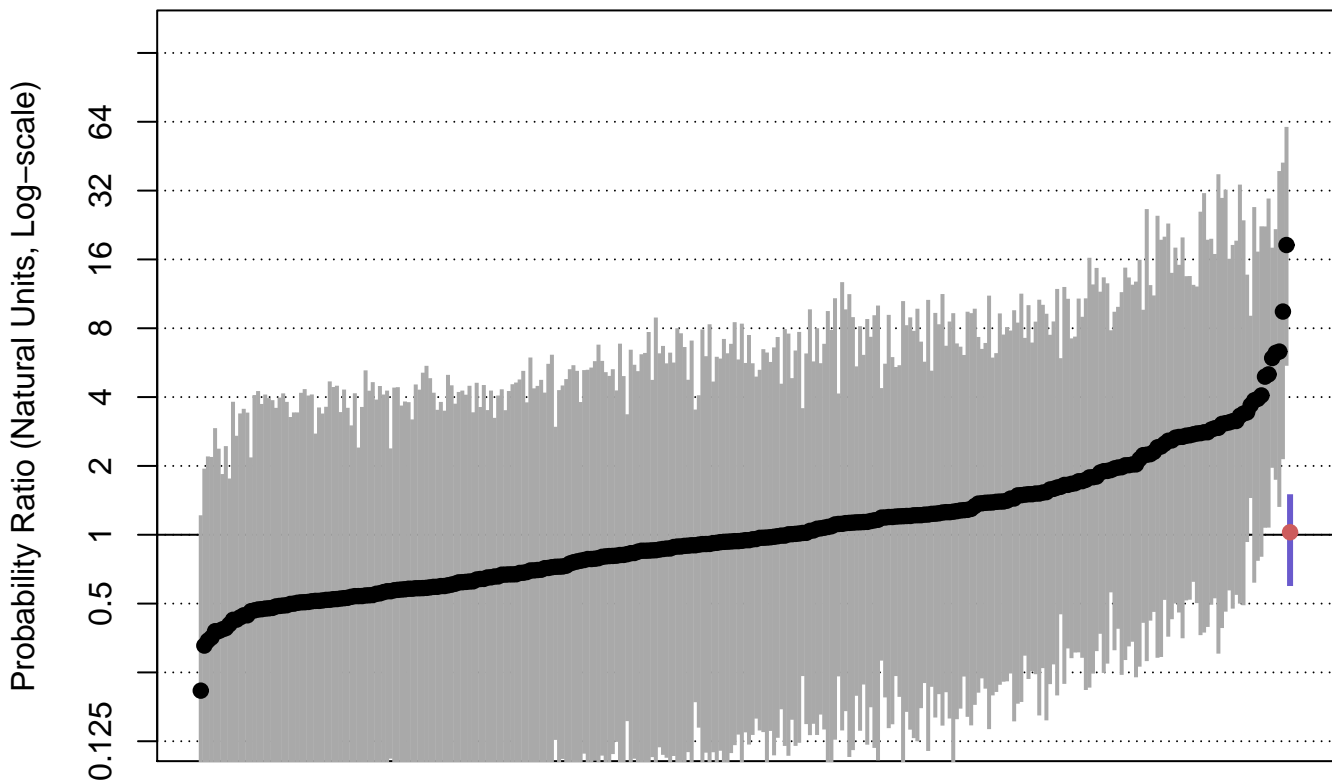

County-Level Ratio of Probabilities of Being Shot By Police: Black-Unarmed to White-Armed

Supplement: S2 File — (ZIP) [file pone.0141854.s002.zip › LaTeXBuild/Figures/RR-Cater-BU-WA-eps-converted-to.pdf]

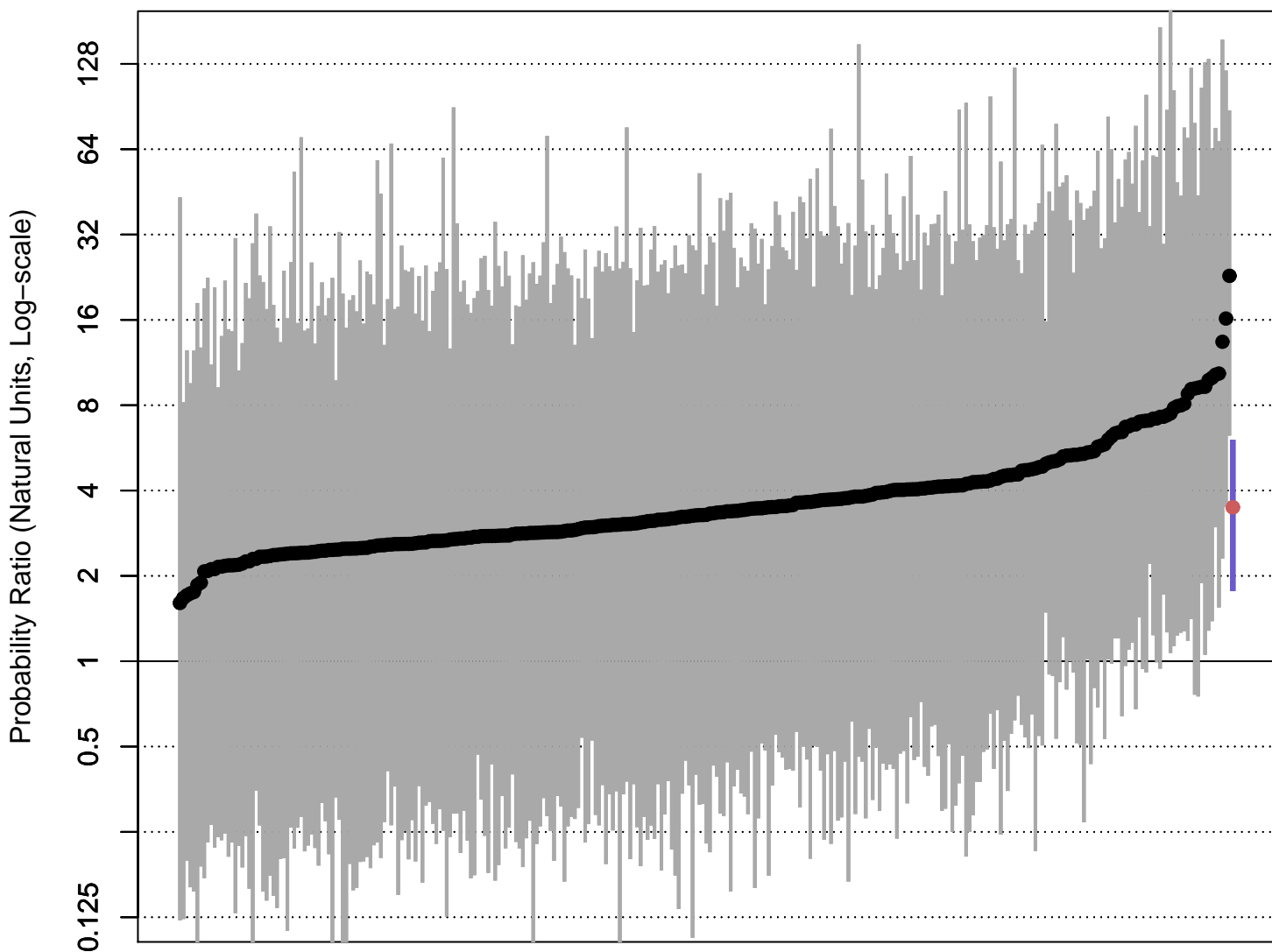

County-Level Ratio of Probabilities of Being Shot By Police: Black-Unarmed to White-Unarmed

Supplement: S2 File — (ZIP) [file pone.0141854.s002.zip › LaTeXBuild/Figures/RR-Cater-BU-WU-eps-converted-to.pdf]

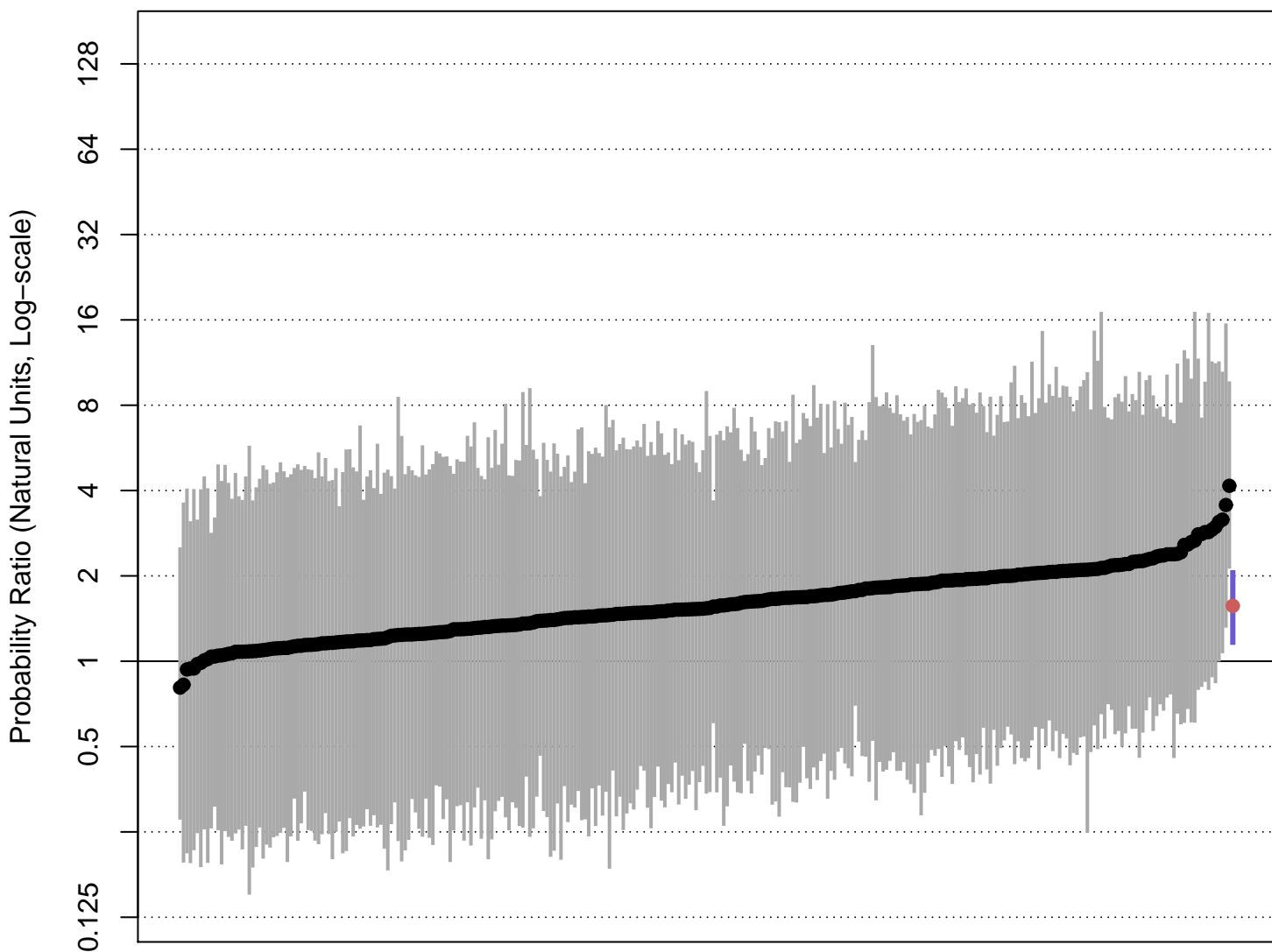

County-Level Ratio of Probabilities of Being Shot By Police: Hispanic-Armed to White-Armed

Supplement: S2 File — (ZIP) [file pone.0141854.s002.zip › LaTeXBuild/Figures/RR-Cater-HA-WA-eps-converted-to.pdf]

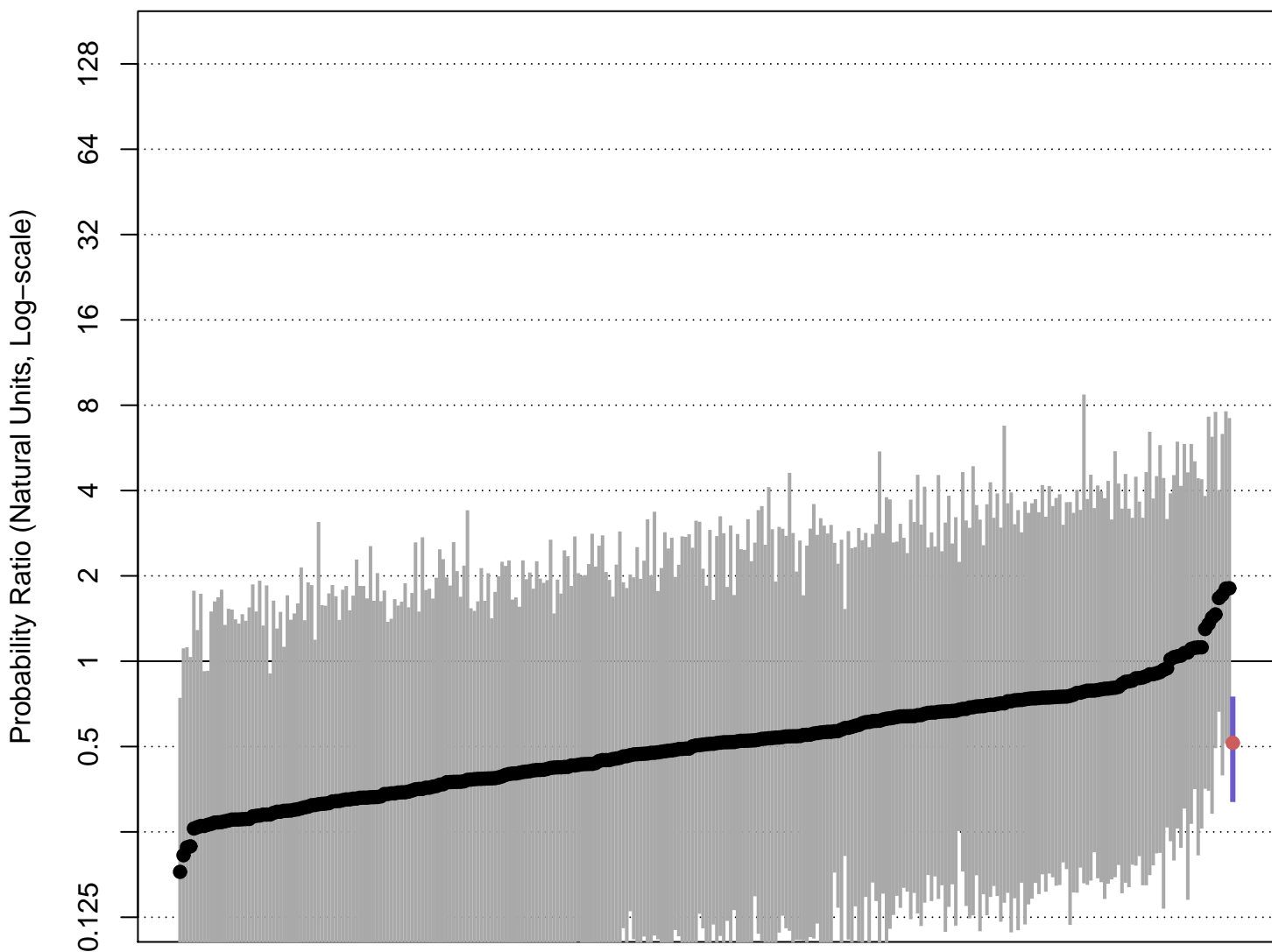

County-Level Ratio of Probabilities of Being Shot By Police: Hispanic-Unarmed to White-Armed

Supplement: S2 File — (ZIP) [file pone.0141854.s002.zip › LaTeXBuild/Figures/RR-Cater-HU-WA-eps-converted-to.pdf]

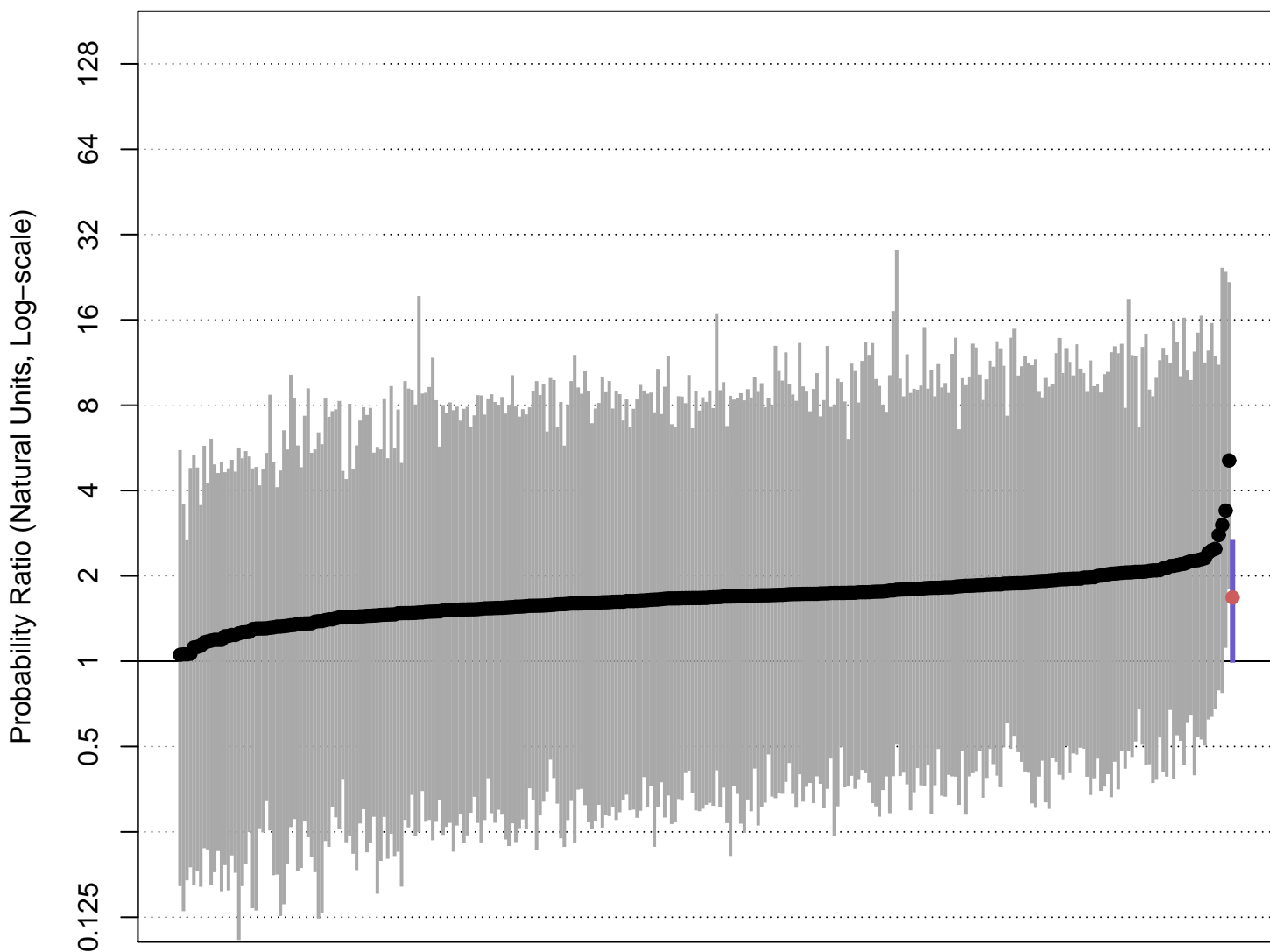

County-Level Ratio of Probabilities of Being Shot By Police: Hispanic-Unarmed to White-Unarmed

Supplement: S2 File — (ZIP) [file pone.0141854.s002.zip › LaTeXBuild/Figures/RR-Cater-HU-WU-eps-converted-to.pdf]

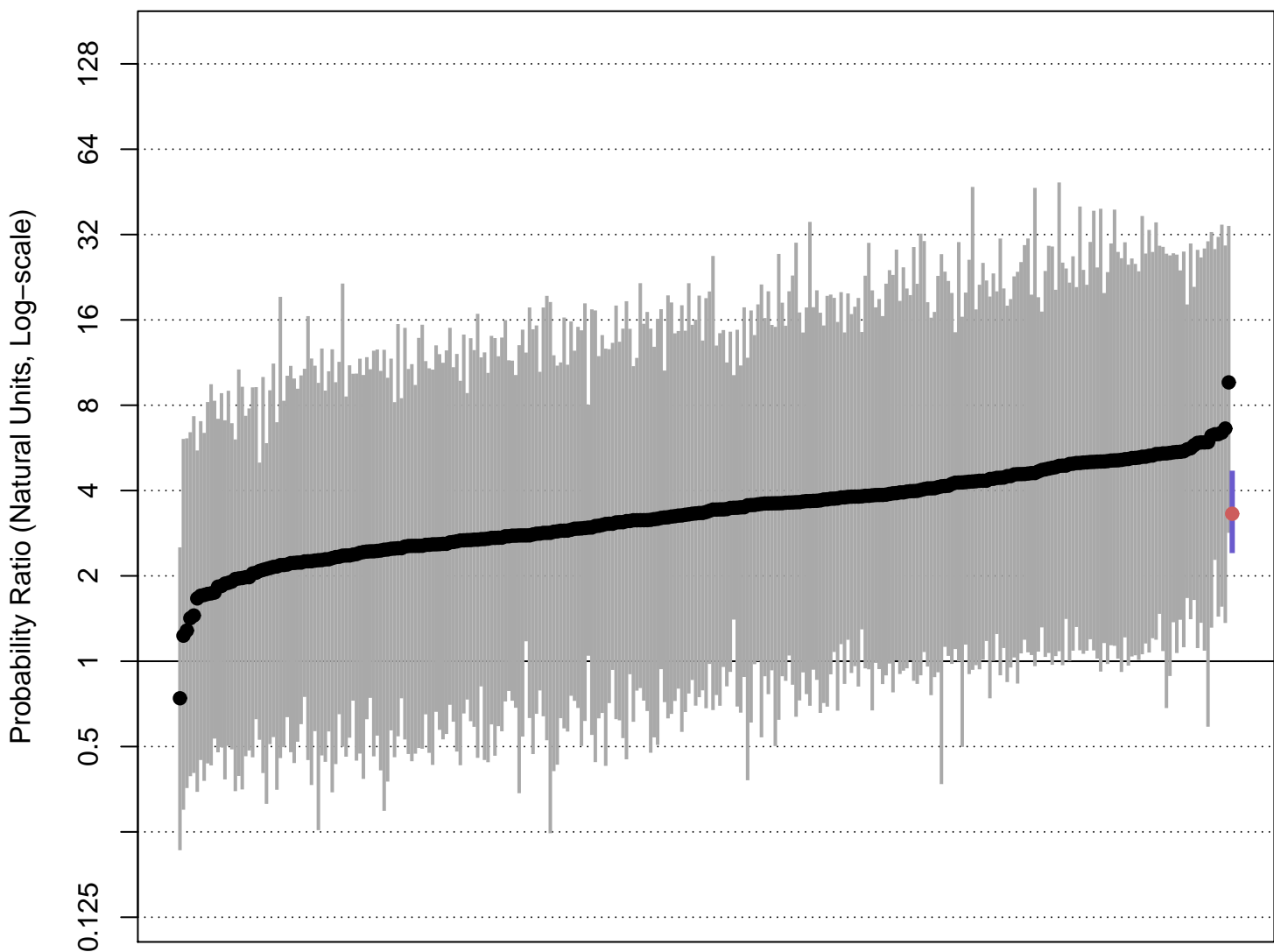

County-Level Ratio of Probabilities of Being Shot By Police: White-Armed to White-Unarmed

Supplement: S2 File — (ZIP) [file pone.0141854.s002.zip › LaTeXBuild/Figures/RR-Cater-WAU-eps-converted-to.pdf]

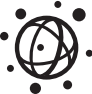

**PLOS**

**SUBMISSION**

Supplement: S2 File — (ZIP) [file pone.0141854.s002.zip › LaTeXBuild/PLOS-submission-eps-converted-to.pdf]
